# Text Mining Analysis of Q-Grader Sensory Descriptors in Specialty Coffee Under Accelerated Storage Conditions

**DOI:** 10.3390/foods15152756

**Published:** 2026-08-05

**Authors:** Frank Fernandez-Rosillo, Lenin Quiñones-Huatangari, Jonathan Alberto Campos Trigoso, Eliana Milagros Cabrejos-Barrios, Segundo G. Chavez, César R. Balcázar-Zumaeta

**Affiliations:** 1 Grupo de Modelamiento y Simulación de Procesos en la Industria Alimentaria (MOSIPRIA), Instituto de Investigación de Ciencia de Datos (INSCID), Universidad Nacional de Jaén (UNJ), Carretera Jaén—San Ignacio KM 24, Cajamarca 06801, Peru; frank_fernandez@unj.edu.pe (F.F.-R.); eliana_cabrejos@unj.edu.pe (E.M.C.-B.); 2 Instituto de Investigación en Ciencia de Datos e Inteligencia Artificial, Facultad de Ingeniería Zootecnista, Biotecnología, Agronegocios y Ciencia de Datos, Universidad Nacional Toribio Rodríguez de Mendoza de Amazonas, Chachapoyas 01001, Peru; lenin.quinones@untrm.edu.pe (L.Q.-H.); jonathan.campos@untrm.edu.pe (J.A.C.T.); 3 Instituto de Investigación para el Desarrollo Sustentable de Ceja de Selva (Indes-Ces), Universidad Nacional Toribio Rodríguez de Mendoza de Amazonas, Chachapoyas 01001, Peru; segundo.quintana@untrm.edu.pe; 4 Instituto de Investigación, Innovación y Desarrollo para el Sector Agrario y Agroindustrial (IIDAA), Facultad de Ingeniería y Ciencias Agrarias, Universidad Nacional Toribio Rodríguez de Mendoza de Amazonas, Chachapoyas 01001, Peru

**Keywords:** natural language processing, packaging, SCA cupping, specialty coffee, text mining, topic modeling

## Abstract

Sensory evaluation is the reference method for assessing specialty coffee quality; however, the descriptive narratives generated by certified Q Arabica Graders remain an underutilized source of information. This study developed an integrated analytical framework combining conventional sensory evaluation with natural language processing (NLP) to characterize the evolution of specialty coffee quality during accelerated storage under different packaging systems. Green and roasted coffee stored in eight packaging configurations were subjected to accelerated storage at 40, 50, and 60 °C, and sensory evaluations were performed according to the Specialty Coffee Association protocol. Textual sensory descriptions were analyzed using descriptor frequency analysis, term frequency–inverse document frequency (TF–IDF) weighting, co-occurrence networks, topic modeling, and topic prevalence analysis. The results demonstrated that the evaluated packaging–product configurations (PPCs), together with storage temperature, influenced the sensory stability of specialty coffee under accelerated storage conditions. Vacuum packaging and multilayer laminated bags more effectively preserved desirable sensory attributes and higher cup scores, whereas elevated temperatures and coffee grinding accelerated quality deterioration, leading to the progressive replacement of freshness-related descriptors by undesirable storage-related sensory characteristics. The combined application of multiple text-mining approaches consistently revealed systematic semantic changes in sensory perception that complemented conventional cup scores and provided a more comprehensive characterization of quality evolution during storage. These findings demonstrate that integrating conventional sensory evaluation with natural language processing transforms expert sensory narratives into reproducible quantitative information, providing a reproducible analytical framework for the objective characterization and comparison of sensory changes during accelerated storage of specialty coffee.

## 1. Introduction

Specialty coffee is distinguished by its exceptional sensory quality, which determines its commercial value and consumer acceptance. The sensory profile of specialty coffee results from the interaction of multiple factors throughout the production chain, including cultivation, post-harvest processing, roasting, and packaging, each contributing to the development of characteristic attributes such as aroma, flavor, acidity, body, sweetness, and overall balance [1]. Consequently, sensory evaluation has become the reference method for assessing coffee quality, with the Specialty Coffee Association (SCA) cupping protocol providing a standardized framework for the evaluation of these attributes [2]. As consumer demand for high-quality coffees with distinctive sensory profiles continues to grow, maintaining consistent sensory quality has become increasingly important for producers, exporters, roasters, and the specialty coffee industry as a whole [3].

Maintaining the characteristic sensory profile of specialty coffee throughout storage remains a major challenge for producers, exporters, and roasters. Although standardized sensory evaluation provides a robust framework for quantifying coffee quality, storage progressively alters sensory perception through the combined effects of temperature, oxygen exposure, and packaging performance, leading to the gradual loss of desirable attributes and the emergence of undesirable storage-related sensory characteristics [4,5,6,7]. The extent of these changes depends not only on storage conditions but also on factors associated with coffee production and processing, including cultivar, post-harvest practices, and fermentation, all of which contribute to the complexity of the final sensory profile [8,9,10,11,12,13]. Consequently, understanding how storage conditions influence the evolution of sensory descriptors is essential for preserving specialty coffee quality, optimizing packaging strategies, and supporting quality management throughout the coffee supply chain.

Recent advances in text mining have created new opportunities for extracting meaningful information from large collections of unstructured textual data. As a subfield of natural language processing, text mining integrates computational linguistics, statistical analysis, and data mining techniques to identify semantic patterns and transform textual information into structured knowledge [14,15,16,17,18,19]. Although these approaches have been successfully applied across diverse scientific disciplines, their application to standardized sensory narratives remains comparatively limited, particularly in food-quality evaluation, where expert sensory descriptions represent a rich but underutilized source of information.

Within food science, text mining has emerged as a valuable approach for analyzing sensory information, consumer perceptions, and food-quality data, supporting quality assessment and decision-making processes [20]. In coffee research, computational approaches have also been applied to relate chemical composition with sensory quality through metabolomics and machine learning techniques [21,22]. However, these studies have primarily focused on chemical characterization, classification tasks, or consumer-generated content rather than on the standardized sensory narratives produced by certified Q Arabica Graders during controlled storage experiments [23,24,25]. Consequently, the potential of text mining to objectively characterize the evolution of specialty coffee sensory quality from expert cupping narratives remains largely unexplored.

Despite these advances, the systematic application of text mining to the standardized sensory narratives generated by certified Q Arabica Graders during controlled storage experiments remains very limited. This gap is particularly relevant because storage-induced quality deterioration is often reflected initially by gradual changes in sensory descriptor patterns and the emergence of undesirable storage-related vocabulary before substantial declines in overall cup scores become evident. Consequently, the descriptive narratives recorded during Specialty Coffee Association (SCA) cupping sessions represent a rich but largely underutilized source of information that could complement conventional sensory scoring and provide a more comprehensive understanding of specialty coffee quality evolution during storage.

To address this gap, the objective of the present study was to develop and validate an integrated analytical framework combining conventional sensory evaluation with text mining to quantify how accelerated storage conditions influence the sensory language and cup quality of specialty coffee. The framework was applied to cupping narratives generated by six certified Q Arabica Graders from coffee samples stored under different packaging systems and accelerated temperatures. We hypothesized that (H1) increasing storage severity promotes a measurable reduction in freshness-related sensory descriptors and a corresponding increase in storage-related sensory characteristics, as quantified through descriptor frequency, TF–IDF weighting, topic prevalence, and multivariate text-mining analyses, (H2) packaging systems with higher oxygen- and moisture-barrier performance preserve higher cup scores and desirable sensory descriptors while reducing the frequency and semantic prominence of storage-related sensory characteristics throughout accelerated storage, and (H3) Integrating standardized sensory evaluation with complementary text-mining approaches provides reproducible and statistically supported identification of semantic patterns associated with specialty coffee quality evolution during storage. By transforming expert sensory narratives into quantitative information, this integrated approach complements conventional sensory evaluation and provides an objective and reproducible framework for quantitative characterization of sensory evolution and comparative evaluation of packaging–product configurations during accelerated storage.

## 2. Materials and Methods

### 2.1. Experimental Workflow

**Figure 1:**
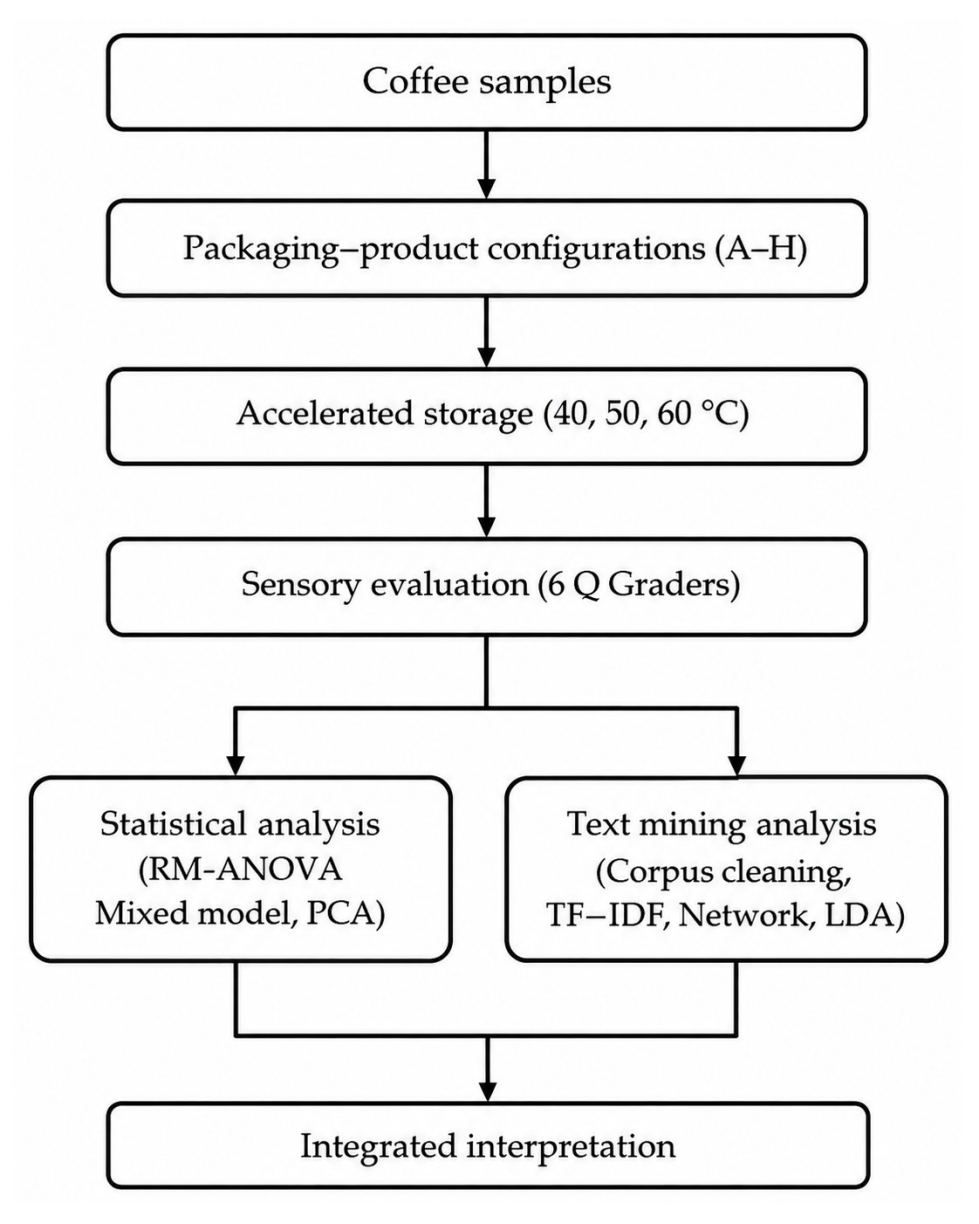
Integrated workflow of the experimental design, sensory evaluation, statistical analysis, and text mining applied to specialty coffee quality assessment.

The study was conducted using an integrated analytical workflow comprising four main stages: experimental design and accelerated storage of coffee under different packaging–product configurations; sensory evaluation by a panel of six certified Q Arabica Graders; statistical analysis of the quantitative sensory data; and text mining applied to the sensory narratives generated during cupping. The overall procedure was adapted from the methodological framework proposed by Zhang et al. [26] and incorporated multivariate analysis and natural language processing techniques to integrate quantitative and qualitative information (Figure 1).

### 2.2. Coffee Samples and Accelerated Storage Experiment

#### 2.2.1. Coffee Samples

The coffee used was a commercial blend consisting of 50% Geisha, 25% Caturra, and 25% Pache (Blend Gourmet Organic Green Gold Coffee). Approximately 160 kg of coffee was used to prepare the various experimental configurations. All samples were obtained from the same production lot to minimize variability associated with origin and processing.

#### 2.2.2. Packaging–Product Configurations

To investigate the combined effects of packaging system and coffee presentation during accelerated storage, eight packaging–product configurations were established. These configurations included green coffee beans, roasted coffee beans, and roasted ground coffee packed using different packaging materials and sealing systems. Each configuration represented a unique combination of coffee presentation and packaging type, allowing the influence of storage conditions to be evaluated across commercially relevant products. For clarity and consistency throughout the statistical and text-mining analyses, each experimental configuration was identified using the nomenclature A–H, as summarized in Table 1.

Hereafter, each experimental unit is referred to as a packaging–product configuration (PPC) for each of the eight experimental combinations (A–H).

#### 2.2.3. Preparation of Packaging–Product Configurations

##### PPC A–C: Green Coffee Beans

Configurations A–C consisted of green coffee beans packaged under three commercial storage systems: tocuyo bags (PPC A), bilaminated aluminum bags (PPC B), and vacuum-sealed Ecotac bags (PPC C). Vacuum packaging was performed using a Grondoy EVE vacuum packaging machine (Grondoy, Jinhua, Zhejiang, China). Each package contained 200 g of green coffee beans. After accelerated storage, the samples were roasted using a PROBAT-WERKE BRZ ×2 roaster (PROBAT SE, Emmerich am Rhein, Germany) at an initial temperature of 185 °C and a final temperature of 190 °C for 15 min. The roasted beans were allowed to rest for 10 min and subsequently ground using a Mahlkönig BRZ2 grinder (Mahlkönig GmbH & Co. KG, Hamburg, Germany). For sensory evaluation, 11.55 g of ground coffee was prepared using the manufacturer’s grind setting level 6, corresponding to the medium grind recommended for the SCA cupping protocol.

##### PPC D: Vacuum-Packed Roasted Coffee Beans

Configuration D consisted of roasted coffee beans vacuum-packed in Ecotac bags (200 g per package) using the same Grondoy EVE vacuum packaging machine (Grondoy, Jinhua, Zhejiang, China). Prior to packaging, coffee was roasted in a Probat P12-2 roaster (PROBAT Inc., Memphis, TN, USA) under a medium roast profile, using an initial temperature of 185 °C and a final temperature of 210 °C for 10.5 min. Roasted beans were allowed to rest for 12 h before packaging. After accelerated storage, the beans were ground using a Mahlkönig BRZ2 grinder (Mahlkönig GmbH & Co. KG, Hamburg, Germany), and 11.55 g of coffee was used for each sensory evaluation.

##### PPC E–H: Roasted Ground Coffee

Configurations E–H consisted of roasted and ground coffee packaged under four commercial systems: cardboard box (PPC E), bilaminated bag without degassing valve (PPC F), bilaminated bag with degassing valve (PPC G), and trilaminated bag with degassing valve (PPC H). Coffee was roasted in a Probat P12-2 roaster (PROBAT Inc., Memphis, TN, USA) under the same medium roast conditions described above and allowed to rest for 12 h. The roasted coffee was ground using a Ditting KFA1403 industrial grinder (Ditting Maschinen AG, Bachenbülach, Switzerland) using the manufacturer’s grind setting level 5, corresponding to the medium grind recommended for the SCA cupping protocol. Ground coffee (200 g per package) was sealed using a Ditting FR-900 heat sealer (Ditting Maschinen AG, Bachenbü-lach, Switzerland).

#### 2.2.4. Accelerated Storage Conditions

All packaging–product configurations were subjected to accelerated storage in FAITHFUL GX-45BE incubators (Faithful Instrument Co., Ltd., Ningbo, Zhejiang, China) maintained at constant temperatures of 40, 50, and 60 °C. Samples were randomly distributed among incubators and identified using individual codes before storage. Five sampling points were established for each temperature to evaluate sensory quality deterioration over time. Samples stored at 40 °C were evaluated on days 0, 3, 6, 9, and 12; those stored at 50 °C on days 0, 2, 4, 6, and 8; and those stored at 60 °C on days 0, 1, 2, 3, and 4. At each sampling point, the corresponding packages were removed from storage and prepared for sensory evaluation according to the procedures described above.

For each packaging–product configuration (PPC), storage temperature, and sampling stage, three independent packages were prepared before the beginning of the experiment as independent experimental replicates and randomly assigned to the corresponding accelerated storage conditions. Each package was opened only once at its designated sampling time following a destructive sampling strategy. During each cupping session, the six certified Q Arabica Graders independently evaluated all three replicate packages according to the SCA protocol. For each grader, the sensory scores obtained from the three independent package replicates were averaged to obtain a single representative score for each experimental condition prior to statistical analysis. Consequently, the inferential analyses were performed using grader-level mean values derived from three independent experimental replicates rather than repeated evaluations of a single package.

### 2.3. Sensory Evaluation: Determination of the Cup Profile of Specialty Coffee

Sensory analysis was performed by six certified Q Arabica Graders following the Specialty Coffee Association (SCA) cupping protocol, adapted from Louzada et al. [27]. The panel evaluated fragrance/aroma, flavor, aftertaste, acidity, body, balance, uniformity, clean cup, sweetness, defects, and overall impression using the official SCA score sheet. Each evaluation was performed using 11.55 g of roasted and ground coffee infused with 150 mL of water at 95 °C in 210 mL cupping bowls. Samples were randomly coded before evaluation to minimize identification bias.

To evaluate panel consistency, inter-rater reliability was assessed using Cronbach’s alpha, intraclass correlation coefficients [ICC(2,1), ICC(2,6), ICC(3,1), and ICC(3,6)], and Kendall’s coefficient of concordance (W). Single-measure coefficients describe the reliability of an individual grader, whereas average-measure coefficients describe the reliability of the six-grader panel meaning.

The coffee was classified according to the final total score according to the SCA, as shown in Table 2, where coffee loses its specialty status with a score below 80 points and is unfit for consumption if the score is lower.

### 2.4. Statistical Analysis of Sensory Data

The sensory dataset comprised repeated evaluations of eight packaging–product configurations (PPCs) performed by six certified Q Arabica Graders under three accelerated storage temperatures and multiple storage periods. Descriptive statistics were first calculated for all quantitative sensory variables and final cup scores. Results are presented as means ± standard deviations (SD), together with their corresponding 95% confidence intervals (95% CI), to summarize the central tendency and variability of the sensory responses.

To assess the consistency of the sensory panel, inter-rater reliability was evaluated using Cronbach’s alpha (α), intraclass correlation coefficients (ICC), and Kendall’s coefficient of concordance (W). Specifically, ICC(2,1) and ICC(2,k) were calculated using a two-way random-effects model to estimate absolute agreement among individual raters and panel averages, respectively, whereas ICC(3,1) and ICC(3,k) were obtained using a two-way mixed-effects model to evaluate consistency. These complementary indices provided a comprehensive assessment of panel repeatability before subsequent statistical analyses.

Panel reliability was evaluated using the overall specialty coffee cup scores independently assigned by the six certified Q Arabica Graders. Reliability statistics were calculated using the complete sensory dataset, pooling all packaging–product configurations, storage temperatures, and storage stages, because the objective was to assess the overall inter-rater agreement of the sensory panel across the full experimental design rather than within individual experimental subsets. Accordingly, Cronbach’s alpha, intraclass correlation coefficients, and Kendall’s coefficient of concordance quantified the consistency of the panel in evaluating the complete set of experimental conditions prior to the subsequent inferential analyses.

The experimental unit was the individual coffee package. For each packaging–product configuration, storage temperature, and sampling stage, three independent packages were prepared as experimental replicates. During each cupping session, each certified Q Arabica Grader independently evaluated the three replicate packages according to the SCA protocol. For each experimental condition, the scores assigned by each grader to the three independent package replicates were averaged to obtain one representative sensory value prior to inferential statistical analyses. Consequently, all inferential statistical analyses were performed using grader-level mean scores derived from three independent experimental replicates rather than individual package evaluations. The effects of packaging–product configuration, storage temperature, and storage stage on specialty coffee quality were evaluated using repeated-measures analysis of variance (RM-ANOVA). To account for the repeated observations collected across storage periods, the primary response variable for the repeated-measures analysis was the change in cup score relative to the corresponding day-0 evaluation within each storage temperature, thereby accounting for the different sampling schedules adopted at 40, 50, and 60 °C. The statistical model included packaging–product configuration (PPC), storage temperature, storage stage, and their interactions as fixed effects. Statistical significance was established at *p* < 0.05. Effect sizes were quantified using partial eta squared (η^2^p), which was interpreted according to conventional thresholds for small, medium, and large effects. Whenever significant differences were detected, pairwise comparisons were performed using Holm-adjusted post hoc tests to control the family-wise error rate.

To further investigate the multivariate structure of sensory deterioration, principal component analysis (PCA) was performed using the ten quantitative sensory attributes defined by the Specialty Coffee Association (SCA). Prior to analysis, all variables were standardized by z-score transformation to eliminate scale differences among attributes. Principal component loadings, explained variance, and sample scores were examined to identify the dominant sources of sensory variation and to visualize relationships among packaging–product configurations during accelerated storage.

Because repeated sensory evaluations were performed by multiple Q Arabica Graders, linear mixed-effects models (LMMs) were additionally fitted as a sensitivity analysis to evaluate the robustness of the RM-ANOVA results. In these models, packaging–product configuration, storage temperature, and storage stage were considered fixed effects, whereas the individual Q Arabica Grader was included as a random intercept to account for between-rater variability. The variance components and conditional intraclass correlation coefficient (ICC) were estimated to quantify the proportion of variability attributable to the sensory panel. Models were estimated by maximum likelihood, and model fit was assessed using the log-likelihood (LogLik), Akaike Information Criterion (AIC), and Bayesian Information Criterion (BIC).

All statistical analyses were performed using Python version 3.10 (Python Software Foundation, Wilmington, DE, USA). Data manipulation and preprocessing were conducted using pandas (version 2.2.2), numerical computations were performed with NumPy (version 1.26.4), inferential statistical analyses were carried out using SciPy (version 1.13.1) and statsmodels (version 0.14.2), multivariate analyses were implemented with scikit-learn (version 1.5.0), and graphical visualizations were generated using Matplotlib (version 3.9.0).

### 2.5. Text Preprocessing and Corpus Normalization

For each combination of packaging–product configuration (PPC), storage temperature, and storage stage, six certified Q Arabica Graders independently generated sensory descriptions following the Specialty Coffee Association (SCA) cupping protocol. To construct the text-mining corpus, the six narratives corresponding to the same experimental condition were concatenated in their original form into a single document without consensus editing, weighting, or selection of descriptors. Consequently, each document represented one experimental condition and integrated the complete vocabulary contributed independently by all six evaluators. This procedure resulted in a corpus comprising 120 condition-level documents (8 PPCs × 3 storage temperatures × 5 storage stages). Before preprocessing, the concatenated corpus contained 3861 word tokens and 207 unique lexical terms.

Text preprocessing was performed to reduce linguistic variability while preserving descriptors with sensory relevance. Because the original narratives contained terms in both Spanish and English, Spanish descriptors were translated into English to produce a linguistically consistent corpus. Translation was conducted at the descriptor level rather than through unrestricted automatic translation to avoid altering the sensory meaning of culturally specific terms.

The translation and lexical normalization procedures were supervised by the lead Q Arabica Grader of the sensory panel, who has more than 30 years of professional experience in specialty coffee sensory evaluation. Descriptor equivalence was established according to its sensory meaning rather than through literal linguistic translation, prioritizing terminology consistent with the Specialty Coffee Association (SCA) Sensory Lexicon. When multiple Spanish descriptors expressed the same sensory perception, the standardized English descriptor was selected by expert consensus based on its sensory equivalence and its conventional use in specialty coffee evaluation. This approach ensured that culturally specific or regional expressions were harmonized without altering their underlying sensory interpretation.

All text was converted to lowercase, and punctuation marks, numerical identifiers, sample codes, temperature labels, and non-informative special characters were removed. General English stopwords and study-specific administrative terms that did not convey sensory information were excluded. In contrast, sensory descriptors and intensity modifiers defined by the Specialty Coffee Association (SCA) cupping protocol—including body, acidity, bright, high, medium, low, and slightly—were intentionally retained because they constitute meaningful sensory attributes for coffee evaluation and contribute directly to the semantic interpretation of the sensory narratives. The complete list of stopwords removed during preprocessing is provided in the Supplementary Material (Supplementary Table S1).

A controlled bilingual sensory lexicon was subsequently applied to standardize spelling variants, synonyms, regional expressions, and semantically equivalent descriptors. For example, the Spanish terms panela, chancaca, azúcar rubia, and azúcar morena were standardized as brown sugar; cartón and papel were grouped under cardboard; and terms associated with aged or stored coffee, including guardado, vejez, and rancio, were normalized as stale. Similarly, related terms referring to legumes, woody notes, fruits, spices, and sweetness were consolidated using a single standardized descriptor. Context-dependent descriptors presenting potential semantic ambiguity were explicitly disambiguated during lexical normalization. In particular, the term green was assigned either to green coffee when referring to the physical state of unroasted coffee or to green/vegetal when describing herbaceous sensory notes, according to the original context of each sensory narrative. The complete controlled bilingual sensory lexicon, including the context-dependent normalization rules applied to ambiguous descriptors, is provided in Supplementary File S1.

The normalized narratives were tokenized into individual sensory terms. Multiword sensory expressions with a single conceptual meaning were preserved as compound tokens using underscores, such as brown sugar, green apple, and red apple. Relevant bigrams were also identified to retain descriptor combinations that would otherwise lose meaning if analyzed as separate words. Tokens were retained only when they represented recognizable sensory attributes, aroma or flavor notes, mouthfeel characteristics, or deterioration-related perceptions. No stemming was applied because reducing descriptors to word roots could obscure distinctions among sensory terms.

Following translation, cleaning, stopword removal, and lexical normalization, the resulting corpus was converted into document–term matrices for subsequent descriptor-frequency analysis, term weighting, co-occurrence analysis, and topic modeling. Corpus-level statistics, including the numbers of tokens and unique standardized descriptors before and after preprocessing, were calculated to quantify the effect of the normalization procedure.

### 2.6. Text Mining and Topic Modeling

The normalized sensory corpus was subsequently analyzed using an integrated natural language processing (NLP) pipeline that progressively transformed qualitative sensory narratives into quantitative semantic information. The analytical workflow comprised six consecutive stages: (i) descriptor frequency analysis, (ii) TF–IDF-based descriptor weighting, (iii) semantic co-occurrence network analysis, (iv) probabilistic topic modeling using Latent Dirichlet Allocation (LDA), (v) quantitative topic-model validation and optimization, and (vi) statistical association between latent topic prevalence and specialty coffee cup scores. This multistep analytical framework integrated descriptive, semantic, and probabilistic text-mining approaches to characterize the evolution of specialty coffee sensory quality during accelerated storage.

Initially, descriptor frequencies were calculated to identify the most frequently occurring sensory terms across the corpus. Word clouds were generated as an exploratory visualization to provide an intuitive overview of descriptor prevalence, where word size was proportional to the frequency of occurrence within the normalized corpus [28,29]. Although primarily descriptive, this visualization facilitated the identification of dominant sensory characteristics and storage-related defects.

To quantify descriptor importance beyond simple frequency counts, term frequency–inverse document frequency (TF–IDF) scores were computed for all standardized descriptors [30]. Unlike raw frequencies, TF–IDF assigns greater weight to descriptors that are highly representative of specific documents while reducing the influence of terms that appear uniformly throughout the corpus. This approach enabled the identification of descriptors that better discriminated among packaging–product configurations, storage temperatures, and storage stages.

Relationships among descriptors were further investigated through co-occurrence network analysis. A weighted semantic network was constructed from the document–term matrix by connecting descriptors that co-occurred within the same sensory narrative. Edge weights represented the frequency of co-occurrence between descriptor pairs, allowing the identification of semantic clusters associated with positive sensory attributes and storage-induced deterioration.

Latent semantic structures within the sensory narratives were explored using Latent Dirichlet Allocation (LDA), a probabilistic Bayesian topic modeling approach [31] that represents each document as a mixture of latent topics and each topic as a probability distribution over descriptors. Candidate LDA models containing between two and ten topics were independently estimated to determine the most appropriate representation of the corpus [31].

Model selection was performed using a quantitative validation framework that simultaneously considered normalized pointwise mutual information (NPMI) coherence, perplexity, topic diversity, and topic stability estimated across multiple independent model initializations. Candidate LDA models containing between two and ten topics were fitted using identical preprocessing procedures and the same normalized document–term representation to ensure direct comparability among competing solutions. Rather than selecting the optimal model according to a single performance metric, the four validation criteria were jointly considered within a composite validation framework, and the model providing the best overall balance among semantic coherence, stability, diversity, and predictive performance was selected for subsequent interpretation. Complete numerical values for all validation metrics are reported in Supplementary Table S2.

Finally, the prevalence of each latent topic within the condition-level sensory documents was estimated from the selected LDA model and associated with the corresponding mean specialty coffee cup scores using Spearman’s rank correlation coefficient. Because each observation represented one aggregated document corresponding to a unique packaging–product configuration, storage temperature, and storage stage, this correlation analysis was intended as a descriptive assessment of the monotonic association between semantic composition and sensory quality rather than as the primary inferential analysis.

## 3. Results

### 3.1. Evolution of Cup Score During Accelerated Storage

Table 3, Table 4 and Table 5 show the results of the evolution of the sensory profile during accelerated storage. At the beginning of the accelerated storage experiment, all packaging–product configurations (PPCs) obtained mean cup scores within the specialty-coffee range. Initial scores varied from 82.90 ± 0.54 points for roasted ground coffee packaged in a cardboard box (PPC E) to 84.29 ± 0.53 points for vacuum-packed roasted coffee beans (PPC D). Green coffee configurations A, B, and C obtained initial scores of 83.82 ± 0.51, 83.96 ± 0.46, and 83.93 ± 0.43 points, respectively, whereas roasted ground coffee configurations F, G, and H obtained scores of 83.63 ± 0.68, 83.81 ± 0.52, and 83.94 ± 0.43 points, respectively. These baseline values indicated relatively comparable initial sensory quality among the experimental configurations, although PPC E showed a slightly lower initial score.

Cup scores progressively declined during accelerated storage, although the magnitude and temporal pattern of deterioration differed among temperatures and PPCs. At 40 °C, the largest reduction was observed for PPC E, whose score decreased from 82.90 ± 0.54 to 78.04 ± 1.27 points by day 12, representing a loss of 4.86 points and a decline below the 80-point specialty threshold. PPCs F, G, and H also showed marked decreases, reaching final scores of 79.96 ± 1.53, 79.96 ± 1.30, and 79.88 ± 1.25 points, respectively. In contrast, vacuum-packed roasted beans (PPC D) and vacuum-packed green beans (PPC C) retained higher final scores of 82.67 ± 0.30 and 82.75 ± 0.50 points, respectively. Green coffee stored in the bilaminated aluminum bag (PPC B) also maintained specialty status, with a final score of 83.17 ± 0.49 points.

At 50 °C, sensory deterioration was again most pronounced for roasted ground coffee stored in cardboard packaging. PPC E declined from 82.90 ± 0.54 to 79.17 ± 0.82 points at the final evaluation stage. The multilayer configurations F, G, and H retained scores close to 80.8 points, whereas PPCs C and D maintained comparatively higher final values of 82.58 ± 1.25 and 82.75 ± 0.69 points, respectively. PPC B also showed relatively good preservation, ending at 82.88 ± 1.33 points. These descriptive trends indicate that whole-bean or green-coffee presentations, particularly under vacuum or multilayer barrier systems, were more resistant to accelerated sensory deterioration than roasted ground coffee in cardboard packaging.

At 60 °C, cup-score deterioration occurred over the shortest experimental period because the evaluation schedule was compressed to days 0, 1, 2, 3, and 4. Despite the shorter exposure time, PPC E showed a marked reduction from 82.90 ± 0.54 to 79.63 ± 2.06 points. PPC A also exhibited a comparatively large decline, reaching 80.38 ± 1.39 points at the final sampling stage. Conversely, vacuum-packed roasted beans (PPC D) retained the highest final mean score, at 83.46 ± 0.84 points, followed by vacuum-packed green beans (PPC C) at 82.96 ± 0.51 points and green coffee in the bilaminated aluminum bag (PPC B) at 82.88 ± 0.59 points. PPCs F, G, and H remained above 81 points, although all exhibited lower scores than at baseline.

Overall, the descriptive results showed that accelerated storage reduced cup quality across all PPCs, but the rate and extent of decline depended on both coffee presentation and packaging system. Roasted ground coffee packaged in cardboard (PPC E) consistently exhibited the greatest deterioration and was the first configuration to fall below the specialty-coffee threshold. In contrast, vacuum-packed roasted beans (PPC D), vacuum-packed green beans (PPC C), and green beans packaged in bilaminated aluminum bags (PPC B) displayed the greatest sensory stability. The statistical significance and magnitude of these differences are examined in Section 3.3.1 and Section 3.3.2.

### 3.2. Reliability of the Sensory Panel

Before examining the effects of accelerated storage on specialty coffee quality, the consistency of the six certified Q Arabica Graders was evaluated to determine the reliability of the sensory dataset. Panel agreement was assessed using complementary reliability statistics, including Cronbach’s alpha (α), intraclass correlation coefficients (ICC), and Kendall’s coefficient of concordance (W).

The reliability analysis was performed using the overall specialty coffee cup scores assigned independently by the six certified Q Arabica Graders across the complete experimental dataset. The sensory panel exhibited excellent internal consistency, with a Cronbach’s alpha of 0.946, indicating a high level of agreement among the evaluators (Table 6). Similarly, the intraclass correlation coefficients demonstrated strong reproducibility of the sensory scores. For individual graders, the absolute-agreement model yielded ICC(2,1) = 0.705, whereas the average-measure coefficient increased to ICC(2,6) = 0.935, indicating excellent reliability of the six-grader panel mean. Comparable results were obtained using the consistency model, with ICC(3,1) = 0.745 and ICC(3,6) = 0.946.

Kendall’s coefficient of concordance further confirmed a high level of agreement among panelists (W = 0.816, *p* < 0.001), demonstrating that the relative ranking of the coffee samples remained highly consistent across evaluators. Collectively, these results indicate that the sensory panel produced highly reproducible evaluations, thereby supporting the use of panel-average cup scores in all subsequent statistical analyses and natural language processing procedures.

Because the sensory panel demonstrated excellent repeatability and agreement across all complementary reliability metrics, the panel-average cup scores were subsequently used to evaluate the effects of packaging–product configuration, accelerated storage temperature, and storage stage on sensory deterioration using repeated-measures statistical analyses.

### 3.3. Statistical Analysis of Sensory Deterioration

To quantitatively assess the effects of accelerated storage on the sensory quality of specialty coffee, changes in cup scores relative to the baseline assessment were analyzed using a hierarchical statistical approach. First, main effects and interactions were evaluated using repeated-measures analysis of variance (RM-ANOVA). Subsequently, the magnitude of these effects was interpreted using effect sizes (partial η^2^), the multivariate structure was explored using principal component analysis (PCA), and, finally, the robustness of the results was verified using mixed linear models.

#### 3.3.1. Repeated Measures ANOVA

Repeated-measures ANOVA revealed significant main effects of packaging–product configuration (F(7,35) = 17.75, *p* < 0.001, η^2^p = 0.780), storage temperature (F(2,10) = 41.56, *p* < 0.001, η^2^p = 0.893), and storage stage (F(3,15) = 46.99, *p* < 0.001, η^2^p = 0.904) on the change in specialty coffee cup score during accelerated storage (Table 7). Among these factors, storage stage exhibited the largest effect size, closely followed by storage temperature, indicating that temporal progression under accelerated storage was the principal driver of sensory deterioration.

Significant interactions were also observed between packaging–product configuration and storage temperature (F(14,70) = 6.13, *p* < 0.001, η^2^p = 0.551), as well as between packaging–product configuration and storage stage (F(21,105) = 2.86, *p* < 0.001, η^2^p = 0.364), demonstrating that the magnitude and progression of sensory deterioration differed among the evaluated configurations. Furthermore, the three-way interaction among packaging–product configuration, temperature, and storage stage remained statistically significant (F(42,210) = 1.62, *p* = 0.015, η^2^p = 0.244), whereas the interaction between temperature and storage stage was not significant (*p* = 0.542).

The three-way interaction among PPC, storage temperature, and storage stage was also statistically significant, indicating that the temporal pattern of sensory deterioration was jointly influenced by both experimental factors. This result demonstrates that no single packaging system performed uniformly across all storage temperatures and sampling stages. Instead, the effectiveness of each packaging–product configuration depended on the combined effects of coffee presentation, storage temperature, and storage duration.

Overall, the repeated-measures ANOVA confirm that sensory deterioration during accelerated storage cannot be explained by storage time alone. Rather, it results from the combined influence of packaging characteristics, coffee presentation, storage temperature, and their interactions, providing a robust statistical basis for the multivariate analyses presented in the following sections.

Although the repeated-measures ANOVA demonstrated statistically significant effects of PPC, storage temperature, and storage stage, statistical significance alone does not indicate the practical importance of these factors. Therefore, effect sizes were subsequently examined to quantify the magnitude of sensory deterioration associated with each packaging–product configuration.

#### 3.3.2. Stratified Analysis by Coffee Presentation

To distinguish the influence of packaging from that of coffee presentation, repeated-measures ANOVA was subsequently performed separately for green coffee (PPCs A–C) and roasted ground coffee (PPCs E–H) (Table 8). This stratified approach eliminated the confounding effect associated with comparing different product presentations and enabled packaging performance to be evaluated within homogeneous coffee categories.

Within the green coffee configurations, cup-score deterioration was significantly affected by packaging configuration (F(2,10) = 40.32, *p* < 0.001, η^2^p = 0.890), accelerated storage temperature (F(2,10) = 4.37, *p* = 0.043, η^2^p = 0.466), and storage stage (F(3,15) = 11.57, *p* < 0.001, η^2^p = 0.698). Among these factors, packaging configuration exhibited the largest effect size, indicating that the choice of storage system was the principal determinant of sensory preservation in green coffee. Furthermore, a significant configuration × storage-stage interaction (F(6,30) = 4.43, *p* = 0.003, η^2^p = 0.470) demonstrated that sensory deterioration progressed at different rates among tocuyo, bilaminated aluminum, and vacuum packaging systems. In contrast, neither the configuration × temperature interaction (*p* = 0.398) nor the three-way interaction (*p* = 0.085) reached statistical significance, indicating that the relative performance of the three packaging systems remained broadly consistent across the evaluated temperatures.

For roasted ground coffee, the relative importance of the experimental factors differed substantially. Storage temperature exerted the strongest influence on cup-score deterioration (F(2,10) = 69.93, *p* < 0.001, η^2^p = 0.933), followed by storage stage (F(3,15) = 23.07, *p* < 0.001, η^2^p = 0.822) and packaging configuration (F(3,15) = 7.08, *p* = 0.003, η^2^p = 0.586). Unlike green coffee, none of the interaction terms reached statistical significance (*p* > 0.05), suggesting that although cardboard, bilaminated, and trilaminated packaging differed in their overall capacity to preserve sensory quality, they followed a similar deterioration trajectory as storage progressed. These findings indicate that, once coffee had been roasted and ground, accelerated temperature became the dominant driver of sensory degradation, whereas packaging primarily influenced the magnitude rather than the pattern of quality loss.

#### 3.3.3. Effect Sizes and Patterns of Sensory Deterioration

**Figure 2:**
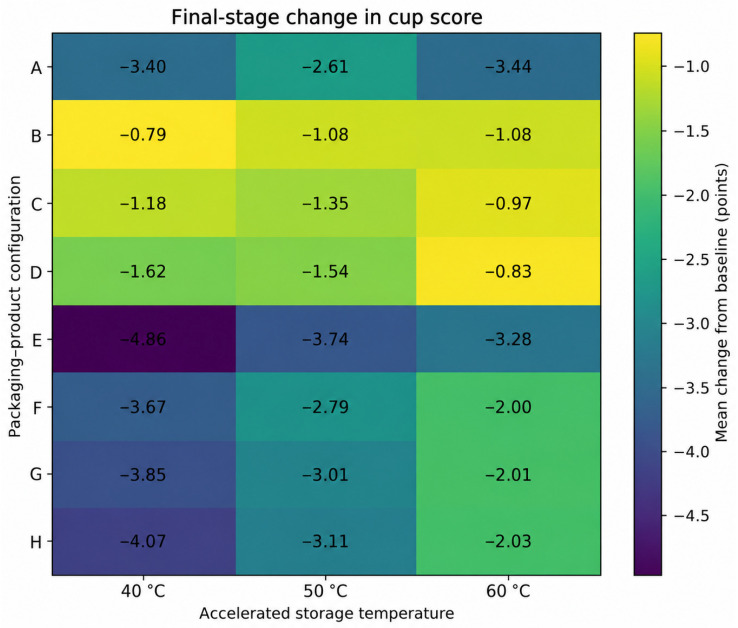
Heatmap showing the final sensory deterioration of specialty coffee after accelerated storage under different packaging–product configurations (PPCs A–H) and storage temperatures (40, 50, and 60 °C). Cell values represent the mean change in cup score (points) relative to the corresponding baseline evaluation (day 0). Negative values indicate reductions in sensory quality after accelerated storage. A = Green coffee–tocuyo; B = Green coffee–bilaminated aluminum; C = Green coffee–vacuum; D = Roasted beans–vacuum; E = Ground coffee–cardboard; F = Ground coffee–bilaminated (no valve); G = Ground coffee–bilaminated (valve); H = Ground coffee–trilaminated (valve). Note: The color scale represents the magnitude of sensory deterioration. Dark purple and blue colors indicate the largest reductions in cup score (greater sensory deterioration), whereas green to yellow colors indicate smaller reductions (better preservation of sensory quality). Each cell displays the corresponding mean change in cup score relative to day 0 for the final storage stage under each temperature.

Although repeated-measures ANOVA identified statistically significant effects of packaging–product configuration, storage temperature, and storage stage, the practical relevance of these factors was better understood by examining their associated effect sizes together with the observed patterns of sensory deterioration (Figure 2).

Partial eta squared (η^2^p) indicated that storage stage (η^2^p = 0.904) and storage temperature (η^2^p = 0.893) exerted the strongest overall influence on changes in cup score, followed by packaging–product configuration (η^2^p = 0.780). These values indicate that temporal progression under accelerated storage and thermal stress accounted for most of the observed sensory deterioration, whereas packaging configuration also contributed substantially to quality preservation. Among the interaction terms, the largest effect corresponded to the interaction between packaging–product configuration and storage temperature (η^2^p = 0.551), demonstrating that the effectiveness of the packaging systems depended on the storage temperature applied. In contrast, the three-way interaction exhibited a comparatively smaller effect size (η^2^p = 0.244), indicating that most variability was explained by lower-order interactions.

The deterioration patterns derived from the mean cup-score reductions further supported these statistical findings (Figure 2). Across all storage conditions, roasted ground coffee packaged in cardboard boxes (PPC E) consistently exhibited the greatest sensory deterioration, particularly at 60 °C, where cup scores declined rapidly throughout storage. Conversely, vacuum-packed green coffee (PPC C), vacuum-packed roasted beans (PPC D), and green coffee stored in bilaminated aluminum bags (PPC B) maintained the highest sensory stability across temperatures and storage stages.

Intermediate deterioration patterns were observed for green coffee stored in traditional tocuyo bags (PPC A) and roasted ground coffee packaged in multilayer barrier materials (PPCs F–H). Although these latter configurations experienced progressive quality losses, deterioration remained considerably lower than that observed for cardboard packaging, particularly under moderate storage temperatures.

Overall, the combination of effect-size analysis and deterioration mapping demonstrates that accelerated storage affects specialty coffee through two complementary mechanisms. Temperature and storage duration determine the overall magnitude of quality loss, whereas packaging systems modulate the extent to which this deterioration is expressed. Consequently, packaging becomes increasingly important as storage conditions become more severe, especially for roasted ground coffee.

#### 3.3.4. Principal Component Analysis

**Figure 3:**
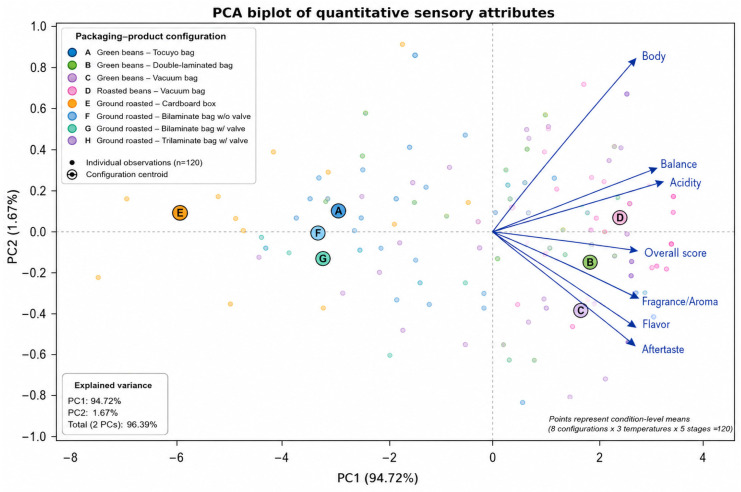
Principal component analysis (PCA) of the ten Specialty Coffee Association sensory attributes evaluated after accelerated storage. Points represent packaging–product configurations (PPCs), whereas vectors correspond to the sensory attributes contributing to sample discrimination. The percentages shown on each axis indicate the proportion of total variance explained by each principal component. Note: Samples located close to each other exhibit similar sensory profiles, whereas greater distances indicate increased sensory dissimilarity. Vector length represents the relative contribution of each sensory attribute to the principal components, and the angle between vectors reflects their correlation. Acute angles indicate positive correlations, right angles indicate weak associations, and opposite directions indicate negative relationships.

Principal component analysis (PCA) was performed using the ten standardized sensory attributes defined by the Specialty Coffee Association to investigate the multivariate structure of sensory changes during accelerated storage. As summarized in Table 9 and illustrated in Figure 3, the first principal component (PC1) explained 94.72% of the total variance, whereas the second principal component (PC2) accounted for an additional 1.67%, resulting in a cumulative explained variance of 96.39%. The third and fourth principal components explained only 1.03% and 0.91% of the total variance, respectively, indicating that the first two components adequately summarized the multivariate sensory dataset.

The PCA biplot (Figure 3) revealed a clear separation among packaging–product configurations according to their degree of sensory deterioration. Configurations B, C, and D clustered near the positive loadings of the SCA sensory attributes, indicating greater preservation of sensory quality, whereas configurations E, F, G, and H were displaced toward the opposite side of PC1, reflecting progressive quality deterioration. Configuration A occupied an intermediate position between these two groups. The observed separation was largely explained by the combined contribution of fragrance/aroma, flavor, aftertaste, acidity, body, balance, sweetness, uniformity, clean cup, and overall impression, indicating that accelerated storage affected the sensory profile as an integrated quality response rather than through isolated attribute changes.

The strong contribution of PC1 indicates that accelerated storage primarily affected the overall sensory quality rather than isolated sensory descriptors. Most SCA attributes exhibited similar loading directions, demonstrating losses in fragrance, flavor, acidity, body, aftertaste, and overall impression occurred simultaneously as storage progressed. This coordinated response suggests that accelerated deterioration was characterized by a generalized decline in sensory quality rather than by selective modification of individual attributes.

PC2 accounted for only a small proportion of the total variability and primarily reflected minor differences among packaging systems under comparable storage conditions. Consequently, most of the sensory discrimination observed in the experiment was captured by the first principal component, confirming the dominant influence of accelerated storage on the global sensory profile.

Overall, the PCA corroborated the repeated-measures ANOVA results by demonstrating that sensory deterioration followed a structured multivariate pattern, with packaging–product configurations preserving higher cup scores clustering separately from those exhibiting pronounced quality losses.

#### 3.3.5. Mixed-Effects Sensitivity Analysis

To assess the robustness of the repeated-measures ANOVA results while explicitly accounting for the hierarchical structure of the sensory evaluations, linear mixed-effects models were fitted using cup score as the response variable; packaging–product configuration (PPC), storage temperature, and storage stage as fixed effects; and Q Arabica Grader as a random intercept. Because only six graders were available as random-effect groups, a parsimonious random-intercept structure was adopted to avoid overparameterization and singular model fits. Models were estimated by maximum likelihood and were used exclusively as sensitivity analyses to determine whether persistent between-grader variability materially influenced the principal interpretation obtained from the repeated-measures analyses.

For the complete dataset (PPC A–H), the estimated grader-level variance was 0.274 and the residual variance was 0.753, resulting in a conditional ICC of 0.235 (Table 10). Thus, approximately 23.5% of the remaining conditional variability after accounting for the fixed experimental factors was attributable to persistent differences among graders, whereas most of the unexplained variability remained at the observation level.

For the green coffee configurations (PPC A–C), the grader-level variance was 0.231 and the residual variance was 0.729, producing a conditional ICC of 0.209. In contrast, the roasted ground coffee configurations (PPC E–H) exhibited a higher grader-level variance (0.555) and a conditional ICC of 0.458, indicating greater evaluator-specific variability in this product category. Nevertheless, these values do not contradict the excellent panel reliability reported in Section 3.2 because the reliability coefficients evaluate agreement among graders, whereas the mixed-effects ICC quantifies the proportion of residual variability attributable to persistent grader-specific differences after accounting for the fixed experimental factors.

The estimated fixed effects were consistent with the principal repeated-measures analysis. In the complete dataset, storage stage remained significantly associated with cup-score deterioration (β = −0.521, SE = 0.032, 95% CI: −0.584 to −0.458, *p* < 0.001), while several packaging–product configurations differed significantly from the reference configuration after accounting for grader-specific intercepts. Similar negative associations between storage stage and cup score were observed for green coffee (β = −0.369, SE = 0.051, 95% CI: −0.470 to −0.269, *p* < 0.001) and roasted ground coffee (β = −0.691, SE = 0.039, 95% CI: −0.767 to −0.614, *p* < 0.001). Complete fixed-effect estimates, confidence intervals, and model-fit statistics are provided in Supplementary Table S3.

Overall, the mixed-effects sensitivity analysis indicated that grader-related variability was limited in the complete and green coffee datasets but more pronounced for roasted ground coffee. Importantly, incorporating Q Arabica Grader as a random intercept yielded fixed-effect patterns consistent with the primary repeated-measures analyses, indicating that the overall patterns of sensory deterioration across the evaluated packaging–product configurations, storage temperatures, and storage stages remained unchanged after accounting for between-grader variability. These results support the robustness of the principal statistical interpretation while also demonstrating the importance of explicitly accounting for evaluator-level variability in roasted ground coffee assessments.

### 3.4. Text Mining Analysis

While the previous sections quantified sensory deterioration through standardized SCA scores, the textual descriptions generated by the certified Q Arabica Graders contain complementary qualitative information that cannot be fully captured by numerical scores alone. Therefore, natural language processing (NLP) and text-mining techniques were applied to characterize the evolution of sensory descriptors, identify latent semantic patterns, and evaluate their relationship with sensory quality during accelerated storage.

#### 3.4.1. Corpus Characterization

The complete sensory corpus comprised 120 sensory descriptions generated by the six certified Q Arabica Graders during the accelerated storage experiment. Prior to text mining, all narratives were translated into English, standardized using a controlled bilingual lexicon, and subjected to tokenization, stop-word removal, punctuation filtering, and descriptor normalization, as described in Section 2.5.

As summarized in Table 11, the original corpus contained 3861 tokens distributed across 120 condition-level documents, corresponding to an average document length of 32.2 tokens per document. After preprocessing and lexical normalization, the corpus comprised 2181 informative tokens, yielding an average document length of 18.2 tokens per document while preserving the original 120 documents. Vocabulary size decreased from 207 to 169 unique descriptors, corresponding to reductions of 43.5% in total tokens and 18.4% in vocabulary size. These preprocessing steps substantially reduced lexical redundancy while preserving the semantic information contained in the sensory narratives.

The resulting corpus constituted the input for all subsequent text-mining analyses, including descriptor frequency analysis, TF–IDF weighting, descriptor co-occurrence networks, and latent topic modeling.

The normalized corpus provided a consistent lexical representation of the sensory narratives, enabling reliable quantitative comparisons of descriptor usage across packaging–product configurations and storage conditions. Subsequent analyses therefore focused on identifying the most representative sensory descriptors and their semantic organization within the corpus.

#### 3.4.2. Descriptor Frequency Analysis

The frequency distribution of the normalized sensory descriptors revealed a structured lexical profile that reflected both the desirable sensory characteristics of specialty coffee and the progressive emergence of storage-related defects. As summarized in Table 12, descriptors associated with sweetness, caramelization, floral aroma, and fruity notes dominated the sensory narratives, whereas descriptors related to oxidation and sensory deterioration occurred less frequently but became increasingly prominent under severe storage conditions.

The most frequent descriptor in the normalized corpus was brown sugar (178 occurrences), followed by chocolate (121), carob (103), caramel (101), malt (86), cardboard (84), and floral (81) (Table 12). The predominance of these descriptors indicates that sweetness-related and caramelized sensory notes remained central components of the sensory language used by the certified Q Arabica Graders throughout the accelerated storage experiment. Likewise, descriptors such as honey, orange, nut, plum, and sweet were consistently represented across the corpus, reflecting the persistence of characteristic specialty coffee flavor attributes.

In contrast, descriptors associated with quality deterioration, including stale, legume, astringent, dry, woody, and later oxidized, were less frequent overall but constituted a coherent group of defect-related terms. Their recurrent presence demonstrates that storage-induced defects were not isolated observations, but recurring components of the sensory narratives generated during accelerated storage. Particularly noteworthy is the relatively high frequency of cardboard (84 occurrences), which ranked among the six most frequent descriptors in the entire corpus despite representing a sensory defect. This finding indicates that papery/cardboard notes became sufficiently recurrent to occupy a central position in the sensory vocabulary as storage progressed.

Overall, the descriptor-frequency analysis indicates that accelerated storage did not simply reduce cup scores but progressively modified the lexical composition of the sensory narratives. The vocabulary evolved from descriptors characterizing sweetness, floral aroma, and fruity complexity toward an increasing representation of oxidation-, woody-, and stale-related terms, providing qualitative evidence that complements the quantitative deterioration identified by the repeated-measures ANOVA, PCA, and mixed-effects analyses.

**Figure 4:**
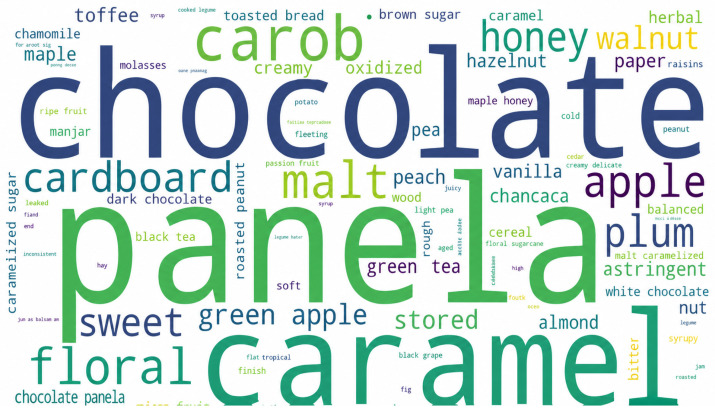
Word cloud representing the normalized sensory descriptors identified in specialty coffee during accelerated storage. Note: The visualization summarizes the complete normalized corpus of sensory narratives produced by six certified Q Arabica Graders across all packaging–product configurations, storage temperatures (40, 50, and 60 °C), and evaluation times. Word size is proportional to descriptor frequency after translation, lexical standardization, stop-word removal, and descriptor normalization.

The overall word cloud (Figure 4) provides a visual representation of the normalized descriptor frequencies summarized in Table 12. The relative size of each term reflects its frequency of occurrence within the corpus, highlighting the predominance of sweetness-related descriptors (e.g., brown sugar, chocolate, caramel, and honey) together with floral and fruity notes. At the same time, defect-related descriptors such as cardboard, stored, paper, and astringent are also clearly represented, illustrating the coexistence of desirable and deterioration-associated sensory attributes throughout the accelerated storage experiment.

Although the global corpus provides an overall representation of the sensory vocabulary, descriptor frequencies may vary according to the accelerated storage temperature. Therefore, temperature-specific lexical profiles were subsequently examined to identify changes in descriptor prominence across storage conditions.

#### 3.4.3. Temperature-Specific Lexical Profiles

**Figure 5:**
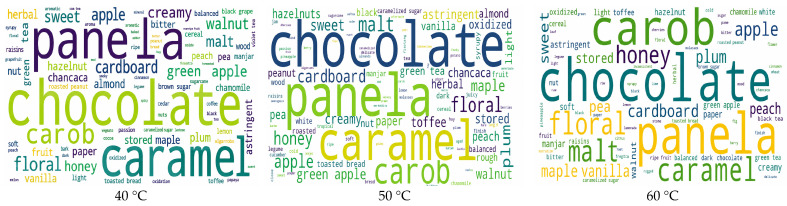
Word clouds showing the normalized sensory descriptors identified at each accelerated storage temperature (40, 50, and 60 °C). Note: Each word cloud was generated independently from the normalized sensory corpus corresponding to each accelerated storage temperature. Text preprocessing included translation into English, lexical standardization, stop-word removal, punctuation filtering, and descriptor normalization. Word size is proportional to the frequency of occurrence of each descriptor within the corresponding temperature-specific corpus. Comparisons among word clouds illustrate changes in descriptor prominence associated with increasing storage temperature rather than absolute differences in descriptor frequency.

To investigate whether accelerated storage temperature influenced the lexical composition of the sensory narratives, separate word clouds were generated for the evaluations performed at 40, 50, and 60 °C (Figure 5). Although the dominant descriptors remained largely consistent across temperatures, noticeable differences in descriptor prominence were observed.

At 40 °C, descriptors such as chocolate, panela, apple, caramel, floral, and malt dominated the corpus, indicating the preservation of sweetness and fruity–floral characteristics. Defect-related descriptors were present but exhibited comparatively lower visual prominence.

At 50 °C, descriptors associated with oxidation and sensory degradation—including astringent, stored, oxidized, and cardboard—became more visually prominent while the relative importance of floral and fruity descriptors decreased. This intermediate profile suggests the onset of perceptible sensory deterioration under accelerated storage.

At 60 °C, sweetness-related descriptors such as chocolate, honey, caramel, and carob remained frequent; however, deterioration-associated descriptors including stored, cardboard, paper, and astringent occupied a larger proportion of the lexical space, indicating that elevated storage temperature modified the qualitative sensory language used by the certified Q Arabica Graders.

Collectively, these lexical shifts indicate that increasing storage temperature modified both the quantitative sensory responses and the lexical composition of the sensory narratives. The greater prominence of defect-related descriptors at higher storage temperatures was consistent with the progressive decline in sensory quality demonstrated by the repeated-measures ANOVA, principal component analysis, and mixed-effects models.

#### 3.4.4. Descriptor Relevance Analysis Using TF–IDF

TF–IDF analysis was used to distinguish descriptors that were merely frequent throughout the corpus from those that were comparatively more representative of storage conditions or packaging–product configurations. At the corpus level, the highest mean TF–IDF values were obtained for brown sugar (0.134), chocolate (0.116), carob (0.106), cardboard (0.103), and caramel (0.102). These results broadly agreed with the absolute-frequency analysis, while also showing that cardboard had substantial discriminatory relevance despite being a defect-related descriptor.

Temperature-specific TF–IDF profiles showed that several sweet and aromatic descriptors remained prominent across the three accelerated storage temperatures, although their relative importance varied. At 40 °C, brown sugar (0.152), chocolate (0.120), and nut (0.112) obtained the highest scores, followed by cardboard (0.106) and floral (0.095). At 50 °C, brown sugar remained the most representative descriptor (0.143), whereas caramel (0.117), chocolate (0.114), cardboard (0.110), and carob (0.109) also showed high relevance. At 60 °C, carob (0.116) and chocolate (0.114) became the leading descriptors, followed by brown sugar (0.107), malt (0.096), and caramel (0.095). Defect-related or quality-deterioration descriptors were also present among the most informative terms, including stale and woody at 40 °C and dry and legume at 60 °C.

Clear differences were also observed among the packaging–product configurations. The green-coffee configurations B and C were primarily characterized by positive floral, fruit, nut, honey, and caramel-related terms. For example, floral showed the highest TF–IDF value in both B (0.127) and C (0.157), while C was additionally characterized by hazelnut (0.153), peach (0.134), and red apple (0.129). The vacuum-packed roasted-bean configuration D was distinguished by honey (0.231), floral (0.180), red apple (0.157), and vanilla (0.142), indicating a predominantly sweet and aromatic lexical profile.

In contrast, the tocuyo-packaged green coffee (A) and the cardboard-packed ground roasted coffee (E) were characterized by a greater contribution of defect-related descriptors. Configuration A showed comparatively high TF–IDF values for green tea (0.228), astringent (0.183), herbal (0.139), dry (0.114), and woody (0.093). Configuration E was strongly differentiated by cardboard (0.284), legume (0.237), oxidized (0.163), stale (0.130), and dry (0.098). In this context, oxidized is reported strictly as a sensory term used in the cupping narratives and should not be interpreted as direct evidence of a chemically measured oxidation process.

The remaining ground roasted-coffee configurations F–H exhibited mixed profiles in which sweet and roasted descriptors coexisted with defect-related terms. Cardboard was the most representative descriptor in F (0.204) and G (0.175), whereas H was mainly characterized by carob (0.146), cardboard (0.135), and stale (0.135). Overall, the TF–IDF analysis complemented the frequency results by identifying the descriptors that most clearly differentiated the experimental conditions, revealing a continuum between retained sweet–aromatic characteristics and increasing prominence of sensory defects during accelerated storage. The complete rankings are provided in Supplementary Table S4.

#### 3.4.5. Descriptor Co-Occurrence Network

**Figure 6:**
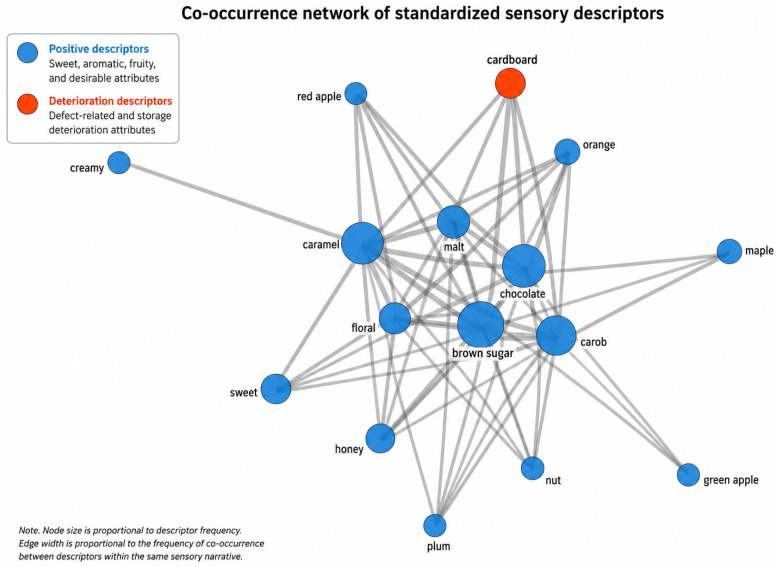
Co-occurrence network of standardized sensory descriptors identified during accelerated storage of specialty coffee. Note: Nodes represent standardized sensory descriptors extracted from the normalized corpus, with node size proportional to descriptor frequency. Edges connect descriptors that co-occurred within the same condition-level sensory narrative, and edge width is proportional to the frequency of co-occurrence. Only recurrent associations exceeding the predefined occurrence threshold were retained to improve network interpretability. The network was generated from the complete corpus comprising all packaging–product configurations, accelerated storage temperatures, and evaluation stages.

To investigate the relationships among sensory descriptors beyond their individual frequencies, a descriptor co-occurrence network was constructed using the standardized corpus (Figure 6). In this network, node size is proportional to descriptor frequency, whereas edge width reflects the frequency with which two descriptors co-occurred within the same sensory narrative. Consequently, the network provides information on the semantic organization of the sensory vocabulary rather than on descriptor abundance alone.

The network revealed a densely connected central structure dominated by descriptors associated with sweet, caramelized, and roasted sensory characteristics. Brown sugar occupied the most central position, showing strong connections with chocolate, caramel, carob, malt, floral, honey, orange, nut, and plum. The high connectivity of these descriptors indicates that they frequently appeared together within the same sensory descriptions, representing the core lexical structure of the specialty coffee sensory profile.

Several fruit- and aroma-related descriptors, including red apple, green apple, orange, floral, and maple, were connected to this central network through multiple shared associations, suggesting that positive sensory attributes were generally described as integrated sensory profiles rather than as isolated characteristics. Likewise, descriptors such as creamy, sweet, and honey exhibited direct connections with the principal sweet–aromatic cluster, reinforcing the coordinated nature of desirable sensory perceptions.

Unlike a fully segregated semantic structure, defect-related descriptors remained integrated within the principal lexical framework of the network. In particular, cardboard exhibited multiple direct connections with brown sugar, chocolate, caramel, and malt, indicating that sensory deterioration was generally described through the coexistence of positive and defect-related attributes rather than by the complete disappearance of desirable sensory descriptors. Rather than forming an isolated semantic cluster, these descriptors became progressively incorporated into the sensory narratives as storage progressed under accelerated conditions, reflecting the gradual evolution of the sensory profile during quality decline.

Overall, the co-occurrence network demonstrates that sensory deterioration was characterized not by the complete replacement of positive descriptors, but by the increasing coexistence of defect-related descriptors within an otherwise sweet–aromatic lexical framework. This semantic organization complements the frequency and TF–IDF analyses by revealing how descriptors were combined by the Q Arabica Graders to describe progressive changes in sensory quality.

#### 3.4.6. Quantitative Validation and Characterization of Latent Topics

**Figure 7:**
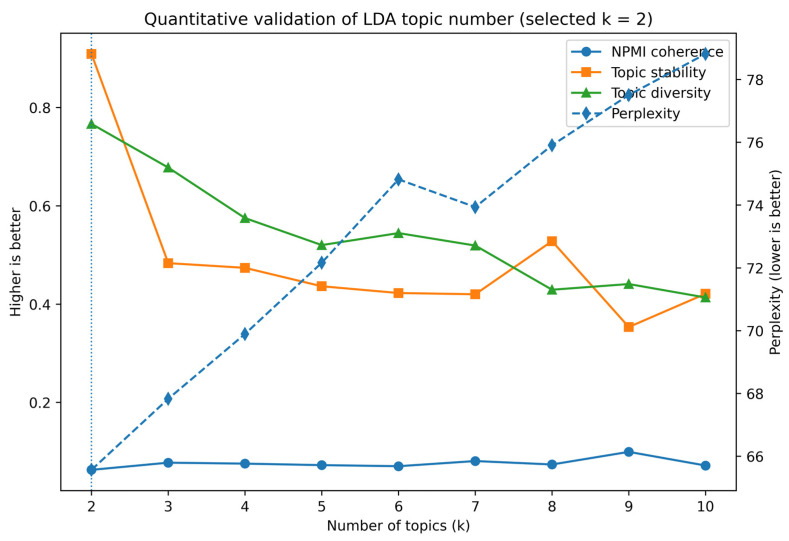
Quantitative validation of the optimal number of latent topics using coherence, stability, diversity, and perplexity metrics. Note: Topic models containing two to ten latent topics were compared using normalized pointwise mutual information (NPMI) coherence, topic stability, topic diversity, and perplexity. Higher coherence, stability, and diversity values indicate improved semantic consistency and reproducibility, whereas lower perplexity indicates better predictive performance. The vertical dashed line indicates the selected two-topic model. The selected model provided the best overall balance among the four complementary validation metrics (Supplementary Table S2).

Latent Dirichlet Allocation (LDA) was applied to identify the principal semantic structures underlying the sensory narratives. Prior to topic interpretation, the optimal number of latent topics was determined by jointly evaluating normalized pointwise mutual information (NPMI) coherence, topic stability, topic diversity, and perplexity across candidate models ranging from two to ten topics (Figure 7). The complete numerical values for all validation metrics are presented in Supplementary Table S2. The two-topic solution was selected because it provided the highest topic stability (0.91), the greatest topic diversity (0.77), and the lowest perplexity (65.7), while maintaining coherence values comparable to those obtained with more complex models. Increasing the number of topics produced only marginal improvements in coherence but was accompanied by reduced stability and diversity together with progressively higher perplexity, indicating lower model interpretability.

Although the absolute NPMI coherence values were relatively low (approximately 0.06–0.10), this behavior is expected for small, highly specialized corpora composed of short sensory narratives in which descriptor sparsity limits co-occurrence frequencies. Consequently, NPMI was not interpreted in isolation but jointly with topic stability, topic diversity, perplexity, and the semantic interpretability of the resulting topics. Under these conditions, the selected two-topic solution was considered the most appropriate representation because it consistently provided the best overall balance among reproducibility, diversity, predictive performance, and meaningful sensory interpretation, rather than maximizing coherence alone.

The selected two-topic solution should therefore be interpreted as a parsimonious semantic representation of the dominant lexical gradient present in the sensory corpus rather than as an exhaustive decomposition of all latent semantic structures. Topic 1 was characterized by descriptors including brown sugar, chocolate, caramel, carob, malt, together with defect-related terms such as cardboard, stale, and woody. Rather than representing an exclusively deteriorated sensory profile, this topic reflected a transitional lexical structure in which desirable sweet–roasted descriptors coexisted with descriptors indicative of sensory quality deterioration.

Topic 2 was dominated by descriptors associated with floral, fruity, and sweet sensory characteristics, including floral, honey, orange, peach, red apple, green apple, creamy, and vanilla. This topic therefore represented a predominantly fresh and aromatic sensory profile, characteristic of coffees with greater sensory preservation.

The coexistence of these two latent semantic structures agrees with the descriptor co-occurrence network (Figure 6), which showed that deterioration-related descriptors remained integrated within the principal lexical framework rather than forming an independent semantic cluster. Consequently, the LDA analysis indicates that the principal semantic organization of the corpus reflected a gradual transition from fresh–aromatic descriptors toward storage-related deterioration descriptors, rather than multiple independent semantic domains.

#### 3.4.7. Relationship Between Topic Prevalence and Sensory Quality

**Figure 8:**
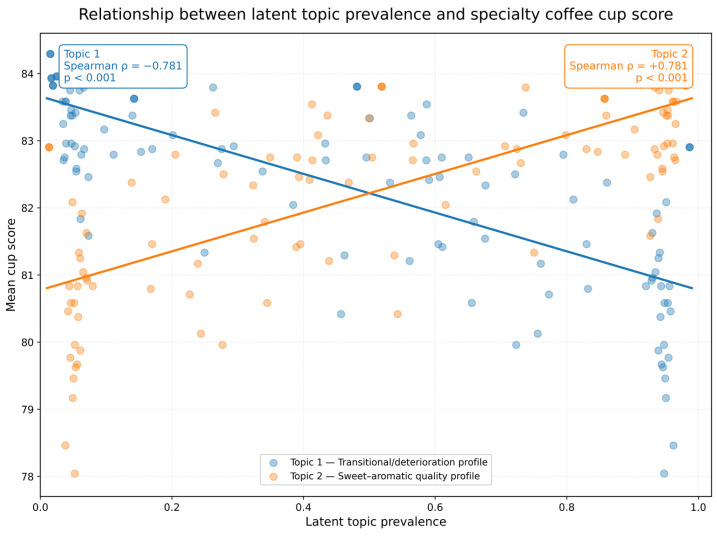
Relationship between latent topic prevalence and mean cup score during accelerated storage of specialty coffee. Note: Each point represents one condition-level sensory narrative. Topic prevalence was estimated using the selected two-topic LDA model. Solid lines indicate linear trends for visualization, whereas the reported Spearman rank correlation coefficients summarizes the monotonic association between topic prevalence and sensory quality. Topic 1 (storage-related deterioration) was negatively associated with cup score (ρ = −0.781, *p* < 0.001), whereas Topic 2 (sweet–aromatic quality) showed an equally strong positive association (ρ = 0.781, *p* < 0.001). Each point represents one aggregated condition-level document obtained by concatenating the six independent sensory narratives corresponding to one packaging–product configuration, storage temperature, and storage stage.

The relationship between latent topic prevalence and sensory quality was explored using Spearman’s rank correlation coefficient as a descriptive measure of monotonic association. Because each observation corresponded to a condition-level document obtained by concatenating the six independent sensory narratives for a given packaging–product configuration, storage temperature, and storage stage, the correlation analysis was intended to summarize the relationship between topic prevalence and mean sensory quality rather than to constitute the primary inferential analysis. The prevalence of the storage-related deterioration topic (Topic 1) exhibited a strong negative monotonic association with mean cup score (Spearman’s ρ = −0.781), whereas the sweet–aromatic quality topic (Topic 2) showed an equally strong positive association (ρ = 0.781) (Figure 8).

The opposite directions of the associations reflect the complementary nature of the two-topic LDA model, in which the prevalence of one topic necessarily increases as the prevalence of the other decreases. Consequently, the correlations should be interpreted as two complementary representations of the same underlying semantic gradient rather than as independent lines of evidence. Within this gradient, Topic 1 was characterized by descriptors associated with storage deterioration, including cardboard, stale, woody, legume, and astringent, together with residual sweet descriptors that progressively persisted during quality decline. Conversely, Topic 2 represented the complementary semantic profile dominated by floral, fruity, caramel, chocolate, honey, and other sweet descriptors characteristic of coffees that maintained higher sensory quality throughout accelerated storage.

The strong monotonic association between the latent semantic gradient represented by the two complementary topics and mean cup score indicates that changes in semantic structure closely paralleled the evolution of conventional sensory quality. Consequently, the text-mining approach not only identified meaningful semantic patterns within the sensory narratives but also quantitatively reflected the progressive loss of cup quality measured by certified Q Arabica Graders.

Together with the repeated-measures ANOVA, principal component analysis, and mixed-effects sensitivity analysis, these findings indicate that accelerated storage altered both quantitative sensory performance and the lexical organization of the sensory descriptions. Collectively, these complementary analytical approaches demonstrate that changes in semantic structure closely tracked the deterioration of specialty coffee quality throughout accelerated storage.

## 4. Discussion

The evaluated packaging–product configurations (PPCs) exhibited marked differences in their ability to preserve specialty coffee quality during accelerated storage, with these differences extending beyond conventional cup scores to encompass the evolution of sensory descriptors, consistent with previous studies highlighting the importance of storage systems in maintaining coffee quality [4,6]. The superior performance of high-barrier multilayer packaging demonstrates that restricting oxygen and moisture transfer effectively delays the physicochemical reactions responsible for aroma degradation and sensory quality loss, supporting earlier observations regarding the protective effects of oxygen-barrier materials on coffee stability [4,7]. These mechanisms are closely associated with the preservation of volatile aroma compounds that define coffee sensory quality [32,33,34]. This trend was consistently supported by repeated-measures ANOVA, principal component analysis, mixed-effects modeling, and natural language processing, all of which consistently identified significant differences among the evaluated packaging–product configurations (PPCs) in terms of sensory stability. Notably, coffees stored in high-barrier packages retained characteristic descriptors such as floral, fruity, honey, caramel, and chocolate for longer storage periods, whereas low-barrier packaging accelerated the appearance of woody, cardboard, stale, and astringent notes, in agreement with previous reports describing the progressive replacement of positive sensory attributes during coffee storage [5,35]. These findings indicate that packaging not only preserves overall cup quality but also contributes to maintaining the sensory identity of specialty coffee throughout storage, preserving the descriptor profiles that characterize high-quality coffees [4,12].

Among the evaluated PPCs, those incorporating high-barrier packaging systems showed greater preservation of sensory quality, consistent with the well-established protective role of oxygen- and moisture-barrier materials reported in previous studies [4,7]. Oxygen exposure accelerates oxidative reactions that progressively deplete aroma-active compounds while promoting the formation of volatile oxidation products associated with woody, cardboard, and stale sensory notes [33,36]. Because the characteristic aroma of specialty coffee depends on a complex balance of volatile compounds, preserving this chemical equilibrium is essential for maintaining desirable sensory attributes such as floral, fruity, honey, caramel, and chocolate notes [32,34,37]. Therefore, the superior performance of high-barrier packaging reflects not only its ability to preserve chemical stability but also its effectiveness in maintaining the sensory identity of specialty coffee throughout storage, highlighting the close relationship between physicochemical preservation and sensory perception.

The present findings further demonstrate that packaging effectiveness should be interpreted as part of a broader interaction involving the physical state of the coffee and storage temperature rather than as an isolated factor. Green coffee, whether stored under vacuum or in multilayer laminated bags, retained a greater proportion of its desirable sensory characteristics than roasted ground coffee exposed to the same accelerated conditions, consistent with previous studies reporting the superior storage stability of unroasted beans [4,6]. In contrast, ground coffee exhibited the fastest sensory deterioration regardless of packaging type, indicating that the protective effect of high-barrier materials becomes progressively limited after grinding. This behavior can be attributed to the substantial increase in exposed surface area, which facilitates oxygen diffusion, accelerates lipid oxidation, enhances volatile compound losses, and reduces the retention of carbon dioxide that naturally delays oxidative degradation in freshly roasted coffee [35,36,38,39]. Elevated storage temperatures further intensified these processes by increasing reaction kinetics and promoting the degradation of aroma-active compounds, thereby accelerating the transition from sweet, floral, and fruity descriptors toward oxidation-related sensory characteristics [6,40,41]. Consequently, preserving specialty coffee quality requires an integrated strategy that simultaneously considers packaging performance, product physical state, and storage conditions throughout the post-roasting supply chain.

Rather than relying exclusively on changes in cup score, the combined interpretation of repeated-measures ANOVA, principal component analysis, mixed-effects modeling, descriptor frequency analysis, TF–IDF, co-occurrence networks, topic modeling, and topic prevalence demonstrated that sensory deterioration is a multidimensional process involving simultaneous changes in quantitative quality scores and the semantic organization of sensory descriptions. This integrated interpretation is consistent with the growing application of text mining and natural language processing for extracting quantitative knowledge from unstructured food-related data [20,30,42]. The close agreement observed among these complementary analytical approaches indicates that modifications in the vocabulary employed by certified Q Arabica Graders accurately reflected the physicochemical changes occurring during storage. Consequently, natural language processing should not be regarded merely as an exploratory text-analysis technique but as a complementary quantitative approach capable of identifying subtle sensory transitions that may precede substantial reductions in cup score, thereby enriching the interpretation of conventional sensory evaluation. Similar trends have recently been reported in food sensory science through the application of text mining and natural language processing to sensory datasets [43,44,45,46,47,48]. Therefore, the integrated analytical framework presented here provides a more comprehensive understanding of specialty coffee quality evolution and represents a promising methodology for future studies focused on storage stability, packaging optimization, and data-driven sensory evaluation.

Although packaging was the primary determinant of specialty coffee preservation, the rate of quality deterioration was strongly modulated by storage temperature and the physical state of the coffee. Increasing storage temperature accelerated the loss of desirable sensory attributes across all packaging systems, although the magnitude of deterioration depended on whether coffee was stored as green beans, roasted beans, or ground coffee [4,6]. Green coffee maintained greater sensory stability because its intact cellular structure provides a natural barrier that limits oxygen penetration and protects intracellular constituents from oxidative degradation. In contrast, roasted coffee, and particularly ground coffee, became progressively more susceptible to quality loss as structural disruption increased oxygen accessibility, promoted volatile compound release, and accelerated oxidative reactions [35,36,38]. Consequently, accelerated storage amplified the intrinsic susceptibility associated with each processing stage, producing distinct deterioration trajectories across coffee matrices that were consistently identified by both conventional sensory evaluation and natural language processing analyses. These findings demonstrate that packaging efficiency is inherently conditioned by the physicochemical characteristics of the product it protects, reinforcing the need to evaluate packaging, product physical state, and storage conditions as interacting components of specialty coffee preservation [34].

Beyond reducing overall cup quality, accelerated storage progressively reshaped the sensory profile of specialty coffee through a sequential replacement of freshness-related descriptors by oxidation-associated sensory attributes. During the early stages of storage, floral, fruity, honey, caramel, chocolate, and brown sugar notes predominated, reflecting the sensory profile typically associated with fresh, high-quality specialty coffees [34]. As storage progressed, particularly under elevated temperatures and in more permeable packaging systems, these descriptors gradually declined while woody, cardboard, stale, dry, and astringent attributes became increasingly prevalent, consistent with previous reports describing sensory deterioration during coffee storage [5,6,35]. This sensory transition reflects the progressive depletion of highly volatile aroma compounds together with the accumulation of oxidation products that alter aroma perception and mouthfeel [32,49,50]. Importantly, deterioration did not occur as an abrupt loss of quality but as a gradual sensory reorganization in which desirable descriptors progressively lost semantic prominence before substantial reductions in cup score became evident. This observation suggests that monitoring changes in the semantic composition of sensory descriptions may provide earlier evidence of quality decline than conventional numerical scores alone, highlighting the dynamic nature of specialty coffee deterioration during storage.

One of the principal methodological innovations of the present study is the application of natural language processing to quantitatively characterize how sensory perception evolves during accelerated storage. Traditional sensory evaluation has historically relied on numerical scores and qualitative tasting notes generated by trained assessors, providing robust information on overall coffee quality. Nevertheless, these descriptive narratives contain substantially richer information than can be represented by cup scores alone, as they capture subtle sensory nuances that are often overlooked in conventional quantitative analyses [34]. By integrating descriptor frequency analysis, TF–IDF weighting, co-occurrence networks, topic modeling, and topic prevalence analysis, the present study demonstrates that textual sensory descriptions can be systematically transformed into quantitative variables describing temporal changes in sensory perception [20,30,42]. Rather than replacing conventional sensory evaluation, natural language processing expands its analytical capacity by objectively identifying semantic patterns, descriptor relationships, and latent thematic structures that are difficult to recognize through manual interpretation alone [48,51]. Consequently, this integrated framework provides a complementary and reproducible approach for understanding the evolution of specialty coffee quality during storage while demonstrating how natural language processing and text mining can complement conventional sensory evaluation through objective and reproducible analysis of sensory narratives.

The robustness of the proposed natural language processing framework is further supported by the remarkable convergence observed across multiple independent analytical approaches. Descriptor frequency analysis consistently identified the progressive replacement of freshness-related sensory descriptors by oxidation-associated attributes, whereas TF–IDF weighting highlighted the descriptors that became increasingly characteristic of specific storage conditions [30]. Likewise, co-occurrence network analysis demonstrated that the semantic organization of sensory descriptors evolved throughout storage, revealing a progressive semantic restructuring of the sensory vocabulary rather than isolated changes in individual terms [20,42]. This interpretation was further reinforced by topic modeling, which condensed complex sensory narratives into coherent latent themes, and by topic prevalence analysis, where the relative importance of these themes closely paralleled the evolution of cup scores [51]. The convergence of these complementary analytical approaches indicates that the semantic transformations observed in the sensory narratives were systematic, reproducible, and directly associated with the progression of coffee quality deterioration rather than representing random variations in descriptive language.

Beyond its methodological advances, the integrated analytical framework developed in this study provides practical opportunities for improving quality management throughout the specialty coffee supply chain. Early identification of semantic changes in sensory descriptions may enable earlier and more informed decision-making regarding packaging selection, storage conditions, and shelf-life optimization before substantial losses in cup quality become evident. This capability is particularly relevant for specialty coffee, where relatively small reductions in sensory quality can markedly influence commercial value and market differentiation [4,34]. Furthermore, integrating conventional sensory evaluation with natural language processing provides coffee producers, exporters, and quality laboratories with an objective strategy for monitoring quality evolution while preserving the detailed information contained in expert sensory narratives [20,42]. As text mining and natural language processing become increasingly integrated into food-quality assessment, incorporating these computational approaches into routine sensory evaluation has the potential to facilitate more reproducible, data-driven, and standardized quality assessment systems across the specialty coffee value chain.

Collectively, the present findings demonstrate that specialty coffee deterioration during storage is best understood as a multidimensional process involving interconnected physicochemical, sensory, and semantic transformations [4,6,34]. While previous studies have primarily assessed storage stability through physicochemical measurements or changes in cup score [41,52], the present work demonstrates that integrating conventional sensory evaluation with natural language processing provides a more holistic representation of quality evolution [20,42]. Rather than viewing sensory narratives as merely qualitative observations, this approach transforms expert language into reproducible quantitative information capable of revealing subtle semantic restructuring in sensory perception that accompanies coffee aging [30]. Consequently, the proposed analytical framework extends current methodologies for specialty coffee quality assessment by integrating complementary sources of sensory information into a unified analytical strategy. This framework establishes a foundation for future applications of natural language processing, text mining, and computational sensory data analysis in food science while illustrating how quantitative analysis of expert sensory narratives can complement conventional sensory evaluation through objective, reproducible, and data-driven characterization of sensory information.

Although the proposed analytical framework proved effective for characterizing the evolution of specialty coffee quality during accelerated storage, the present study was conducted using a single commercial specialty coffee blend obtained from one production lot. This experimental design minimized variability associated with cultivar composition, geographical origin, and post-harvest processing, thereby strengthening the internal validity of the comparisons among storage temperatures and packaging systems. Nevertheless, the findings should not be interpreted as universally applicable to all specialty coffees, since differences in cultivar, origin, processing method, and chemical composition may influence both sensory evolution and descriptor patterns during storage. Future studies should therefore evaluate additional cultivars, geographical origins, processing methods, and commercial blends to assess the broader applicability of the proposed analytical framework under diverse specialty coffee production scenarios and should incorporate parallel conventional storage experiments to establish predictive relationships between accelerated aging and natural storage under commercial conditions. In addition, the experimental design focused exclusively on accelerated storage conditions and did not include a parallel conventional storage control at ambient temperature. Therefore, the present study was designed to compare the relative effects of accelerated storage conditions rather than to establish quantitative conversion relationships between accelerated and natural storage; in addition, because coffee physical state (green, roasted bean, or ground coffee) was intrinsically linked to specific packaging–product configurations, the present design does not permit the independent estimation of packaging and physical-state effects. Consequently, the results should be interpreted at the level of the evaluated PPCs rather than as isolated effects of packaging or coffee physical state.

## 5. Conclusions

The present study demonstrates that text mining can successfully transform Q Arabica Grader sensory narratives into reproducible quantitative information describing the evolution of specialty coffee quality during accelerated storage. By integrating conventional sensory evaluation with natural language processing, the proposed analytical framework revealed distinct patterns of quality evolution among the evaluated packaging–product configurations (PPCs) under different accelerated storage temperatures. Among the evaluated packaging–product configurations (PPCs), those incorporating high-barrier packaging systems, particularly vacuum packaging and multilayer laminated bags, were associated with greater preservation of desirable sensory attributes and higher cup scores, whereas the evaluated PPCs corresponding to roasted ground coffee stored at elevated temperatures were associated with faster sensory deterioration and a progressive replacement of desirable descriptors by storage-related sensory characteristics. Collectively, these findings indicate that specialty coffee deterioration should be understood as a multidimensional process involving interconnected physicochemical, sensory, and semantic transformations rather than as a simple decline in overall cup quality.

Beyond demonstrating the value of text mining for sensory data analysis, this study proposes a reproducible analytical workflow integrating standardized sensory evaluation, text preprocessing, descriptor frequency analysis, TF–IDF weighting, co-occurrence network analysis, quantitatively validated topic modeling, topic prevalence analysis, and multivariate statistical interpretation. This integrated framework provides a practical and transferable strategy for objectively characterizing the evolution of specialty coffee sensory quality and comparing differences among the evaluated packaging–product configurations during accelerated storage through reproducible analysis of expert sensory narratives. By preserving and objectively quantifying the information contained in expert sensory narratives, this approach complements conventional sensory evaluation while improving the reproducibility and interpretability of quality assessment. More broadly, the proposed framework establishes a foundation for future applications of natural language processing, text mining, and data-driven sensory analytics in specialty coffee and other high-value food products. Furthermore, because coffee physical state was intrinsically associated with specific packaging–product configurations, the present experimental design does not permit the independent estimation of packaging and physical-state effects. Accordingly, the conclusions should be interpreted at the level of the evaluated PPCs. Future research should evaluate additional cultivars, geographical origins, post-harvest processing methods, and commercial blends to determine the broader applicability and robustness of the proposed analytical framework across diverse specialty coffee production scenarios.

## Figures and Tables

**Table 1:** Packaging–product configurations evaluated during the accelerated storage experiment.

Configuration	Coffee Presentation	Packaging System
A	Green coffee beans	Tocuyo bag
B	Green coffee beans	Bilaminated aluminum bag
C	Green coffee beans	Vacuum bag
D	Roasted coffee beans	Vacuum bag
E	Roasted ground coffee	Cardboard box
F	Roasted ground coffee	Bilaminated bag (without degassing valve)
G	Roasted ground coffee	Bilaminated bag with degassing valve
H	Roasted ground coffee	Trilaminated bag with degassing valve

Note. A–H: Abbreviation used throughout the manuscript.

**Table 2:** Coffee classification based on the total rating of attributes.

Total Score	Quality	Rating
90–100	Outstanding	Specialty
85–89.99	Excellent	
80–84.99	Very good	
<80	Below specialty quality	Not specialty

**Table 3:** Cup score during storage at 40 °C.

Days	Temperature 40 °C/Relative Humidity 85%							
	Green Coffee Beans			Roasted Coffee Beans	Roasted Ground Coffee			
	A	B	C	D	E	F	G	H
0	83.82 ± 0.512	83.96 ± 0.462	83.93 ± 0.433	84.29 ± 0.529	82.90 ± 0.539	83.63 ± 0.676	83.81 ± 0.524	83.94 ± 0.430
3	82.46 ± 0.954	83.25 ± 0.776	83.58 ± 0.563	83.38 ± 0.440	80.46 ± 1.030	82.38 ± 0.345	82.50 ± 0.474	82.42 ± 0.408
6	81.58 ± 0.683	82.96 ± 0.332	83.38 ± 0.306	82.79 ± 0.714	79.67 ± 1.137	80.92 ± 1.021	81.17 ± 0.769	81.42 ± 0.606
9	81.46 ± 1.279	82.71 ± 0.900	82.79 ± 0.749	82.92 ± 0.605	78.46 ± 1.364	80.13 ± 1.046	80.58 ± 0.876	80.71 ± 0.714
12	80.42 ± 1.310	83.17 ± 0.492	82.75 ± 0.500	82.67 ± 0.303	78.04 ± 1.269	79.96 ± 1.528	79.96 ± 1.298	79.88 ± 1.252

Note. A: Tocuyo bag with green beans. B: Double-laminated aluminum foil bag with green beans. C: Ecotac vacuum bag with green beans. D: Ecotac vacuum bag with roasted beans. E: Pressed cardboard box (ground roasted beans). F: Bilaminate bag without valve with window and zipper (ground roasted). G: Bilaminate bag with degassing valve and zipper (ground roasted). H: Trilaminate bag with degassing valve and zipper (ground roasted). ±: Mean Standard Deviation for six repetitions.

**Table 4:** Cup score during storage at 50 °C.

Days	Temperature 50 °C/Relative Humidity 85%							
	Green Coffee Beans			Roasted Coffee Beans	Roasted Ground Coffee			
	A	B	C	D	E	F	G	H
0	83.82 ± 0.512	83.96 ± 0.462	83.93 ± 0.433	84.29 ± 0.529	82.90 ± 0.539	83.63 ± 0.676	83.81 ± 0.524	83.94 ± 0.430
2	81.83 ± 3.129	83.58 ± 0.376	83.79 ± 0.459	84.04 ± 0.368	81.25 ± 1.140	82.75 ± 0.689	82.71 ± 0.557	82.92 ± 0.465
4	81.33 ± 1.180	82.54 ± 0.914	82.88 ± 0.997	83.08 ± 0.683	79.77 ± 0.965	81.29 ± 1.208	81.54 ± 0.914	81.46 ± 0.765
6	81.33 ± 0.540	82.54 ± 0.679	82.79 ± 0.843	82.83 ± 0.801	79.46 ± 1.840	80.58 ± 1.402	80.83 ± 1.033	80.96 ± 1.042
8	81.21 ± 1.145	82.88 ± 1.330	82.58 ± 1.252	82.75 ± 0.689	79.17 ± 0.816	80.83 ± 1.221	80.79 ± 1.298	80.83 ± 1.262

Note. A: Tocuyo bag with green beans. B: Double-laminated aluminum foil bag with green beans. C: Ecotac vacuum bag with green beans. D: Ecotac vacuum bag with roasted beans. E: Pressed cardboard box (ground roasted beans). F: Bilaminate bag without valve with window and zipper (ground roasted). G: Bilaminate bag with degassing valve and zipper (ground roasted). H: Trilaminate bag with degassing valve and zipper (ground roasted). ±: Mean Standard Deviation for six repetitions.

**Table 5:** Cup score during storage at 60 °C.

Days	Temperature 60 °C/Relative Humidity 85%							
	Green Coffee Beans			Roasted Coffee Beans	Roasted Ground Coffee			
	A	B	C	D	E	F	G	H
0	83.82 ± 0.512	83.96 ± 0.462	83.93 ± 0.433	84.29 ± 0.529	82.90 ± 0.539	83.63 ± 0.676	83.81 ± 0.524	83.94 ± 0.430
1	83.54 ± 0.600	83.75 ± 0.316	83.79 ± 0.292	84.21 ± 0.400	82.08 ± 1.114	83.33 ± 0.516	83.38 ± 0.703	83.42 ± 0.983
2	82.71 ± 0.600	83.42 ± 0.342	83.75 ± 0.524	83.88 ± 0.565	81.04 ± 0.828	82.46 ± 0.557	82.75 ± 0.592	82.96 ± 0.459
3	82.13 ± 0.518	83.08 ± 0.665	83.58 ± 0.540	83.38 ± 0.440	80.58 ± 1.057	82.38 ± 0.862	82.33 ± 0.917	82.04 ± 0.714
4	80.38 ± 1.394	82.88 ± 0.586	82.96 ± 0.510	83.46 ± 0.843	79.63 ± 2.060	81.63 ± 1.358	81.79 ± 1.327	81.92 ± 1.242

Note. A: Tocuyo bag with green beans. B: Double-laminated aluminum foil bag with green beans. C: Ecotac vacuum bag with green beans. D: Ecotac vacuum bag with roasted beans. E: Pressed cardboard box (ground roasted beans). F: Bilaminate bag without valve with window and zipper (ground roasted). G: Bilaminate bag with degassing valve and zipper (ground roasted). H: Trilaminate bag with degassing valve and zipper (ground roasted). ±: Mean Standard Deviation for six repetitions.

**Table 6:** Reliability statistics of the six certified Q Arabica Graders.

Statistic	Estimate	Interpretation
Cronbach’s α	0.946	Excellent internal consistency
ICC(2,1)	0.705	Good absolute agreement (single grader)
ICC(2,6)	0.935	Excellent absolute agreement (panel mean)
ICC(3,1)	0.745	Good consistency (single grader)
ICC(3,6)	0.946	Excellent consistency (panel mean)
Kendall’s W	0.816	Strong concordance
*p*-value (Kendall’s W)	<0.001	Statistically significant agreement

Note. Reliability statistics were calculated using the overall specialty coffee cup scores assigned independently by the six certified Q Arabica Graders across all packaging–product configurations, storage temperatures, and storage stages included in the experiment. The complete dataset was used because the objective was to evaluate overall inter-rater agreement for the sensory panel prior to subsequent inferential analyses. Cronbach’s alpha evaluated internal consistency among graders. Intraclass correlation coefficients (ICC) were calculated using two-way random-effects [ICC(2,1) and ICC(2,6)] and two-way mixed-effects [ICC(3,1) and ICC(3,6)] models. Kendall’s coefficient of concordance (W) quantified agreement in sample ranking among graders.

**Table 7:** Repeated measures ANOVA for changes in specialty coffee cup score during accelerated storage.

Source of Variation	df	F	*p*-Value	Partial η^2^
Packaging–product configuration	7.35	17.75	<0.001	0.780
Storage temperature	2.10	41.56	<0.001	0.893
Storage stage	3.15	46.99	<0.001	0.904
PPC × Temperature	14.70	6.13	<0.001	0.551
PPC × Storage stage	21.105	2.86	<0.001	0.364
Temperature × Storage stage	6.30	0.88	0.542	—
PPC × Temperature × Storage stage	42.210	1.62	0.015	0.244

Note. The response variable corresponds to the change in cup score relative to the baseline evaluation (day 0). Statistical significance was assessed using repeated-measures ANOVA. Partial eta squared (η^2^) was calculated to estimate the magnitude of each effect.

**Table 8:** Stratified repeated-measures ANOVA for changes in specialty coffee cup score within comparable packaging–product groups.

**A. Green coffee (PPC A–C)**				
**Source of variation**	**df**	**F**	***p*-value**	**Partial η^2^**
Packaging–product configuration	2.10	40.32	<0.001	0.890
Storage temperature	2.10	4.37	0.043	0.466
Storage stage	3.15	11.57	<0.001	0.698
PPC × Temperature	4.20	1.07	0.398	0.176
PPC × Storage stage	6.30	4.43	0.003	0.470
Temperature × Storage stage	6.30	1.23	0.319	0.197
PPC × Temperature × Storage stage	12.60	1.72	0.085	0.256
**B. Roasted ground coffee (PPC E–H)**				
**Source of variation**	**df**	**F**	***p*-value**	**Partial η^2^**
Packaging–product configuration	3.15	7.08	0.003	0.586
Storage temperature	2.10	69.93	<0.001	0.933
Storage stage	3.15	23.07	<0.001	0.822
PPC × Temperature	6.30	0.78	0.589	0.136
PPC × Storage stage	9.45	0.57	0.817	0.102
Temperature × Storage stage	6.30	1.13	0.366	0.185
PPC × Temperature × Storage stage	18.90	1.14	0.327	0.186

Note. Partial eta squared (η^2^p) was used as the effect-size measure. Analyses were performed separately for green coffee (PPC A–C) and roasted ground coffee (PPC E–H) to isolate the influence of packaging within comparable product presentations. The common baseline evaluation (day 0) was excluded from the repeated-measures model to avoid duplication across the three temperature-specific storage trajectories.

**Table 9:** Variance explained by the principal components obtained from the standardized SCA sensory attributes.

Principal Component	Eigenvalue	Explained Variance (%)	Cumulative Variance (%)
PC1	9.472	94.72	94.72
PC2	0.167	1.67	96.39
PC3	0.103	1.03	97.42
PC4	0.091	0.91	98.33

Note. Principal component analysis (PCA) was performed using the ten standardized sensory attributes defined by the Specialty Coffee Association (SCA). Eigenvalues correspond to the variance explained by each principal component after variable standardization.

**Table 10:** Variance components estimated from the linear mixed-effects sensitivity models.

Dataset	Grader-Level Variance	Residual Variance	Model ICC	LogLik	AIC	BIC
Complete dataset (PPC A–H)	0.274	0.753	0.235	−741.10	1506.2	1558.47
Green coffee (PPC A–C)	0.231	0.729	0.209	−277.29	568.59	592.22
Roasted ground coffee (PPC E–H)	0.555	0.556	0.458	−332.69	681.38	710.68

Note. Linear mixed-effects models were fitted using cup score as the response variable; packaging–product configuration, storage temperature, and storage stage as fixed effects; and Q Arabica Grader as a random intercept. Models were estimated by maximum likelihood and were included as sensitivity analyses to evaluate whether between-grader variability influenced the primary interpretation obtained from the repeated-measures analyses. Accordingly, the emphasis was placed on variance components, conditional intraclass correlation coefficients (ICC), and model-fit statistics, including the log-likelihood (LogLik), Akaike Information Criterion (AIC), and Bayesian Information Criterion (BIC), whereas complete fixed-effect estimates are reported in Supplementary Table S3. Grader-level variance represents systematic between-evaluator variability remaining after accounting for the fixed experimental factors. Residual variance represents the remaining within-observation variability. The model ICC quantifies the proportion of conditional variability attributable to persistent differences among Q Arabica Graders.

**Table 11:** Summary statistics of the sensory text corpus before and after preprocessing.

Corpus Characteristic	Original Corpus	Normalized Corpus
Documents	120	120
Total tokens	3861	2181
Average document length (tokens/document)	32.2	18.2
Unique descriptors	207	169
Token reduction (%)	—	43.5
Vocabulary reduction (%)	—	18.4

Note. The normalized corpus was obtained after translation into English, lexical standardization using a controlled bilingual lexicon, stop-word removal, punctuation filtering, and descriptor normalization. Document count remained unchanged because preprocessing affected only the lexical representation of the sensory narratives. Average document length was calculated as the total number of tokens divided by the number of condition-level documents.

**Table 12:** Twenty most frequent normalized sensory descriptors identified in the sensory corpus.

Rank	Descriptor	Frequency	Documents-Normalized Frequency
1	brown sugar	178	1.483
2	chocolate	121	1.008
3	carob	103	0.858
4	caramel	101	0.842
5	malt	86	0.717
6	cardboard	84	0.7
7	floral	81	0.675
8	honey	64	0.533
9	orange	57	0.475
10	nut	53	0.442
11	plum	52	0.433
12	sweet	51	0.425
13	stale	42	0.35
14	maple	42	0.35
15	legume	39	0.325
16	red apple	37	0.308
17	green apple	35	0.292
18	creamy	35	0.292
19	vanilla	31	0.258
20	astringent	30	0.25

Note. Frequencies were calculated from the normalized corpus after translation, lexical standardization, stop-word removal, spelling correction, and synonym consolidation. The documents-normalized frequency represents the average occurrence of each descriptor across the 120 sensory narratives included in the corpus.

## Data Availability

The original contributions presented in this study are included in the article/Supplementary Materials. Further inquiries can be directed to the corresponding author.

## Supplementary Materials

The following supporting information can be downloaded at https://www.mdpi.com/article/10.3390/foods15152756/s1. Supplementary File S1 contains the anonymized sensory corpus, including the original sensory narratives, their English translations, and the normalized descriptors used for text mining. Supplementary Table S1 provides the complete stopword list used during text preprocessing. Supplementary Table S2 reports the quantitative validation metrics for candidate LDA models (k = 2–10). Supplementary Table S3 contains the fixed-effect estimates from the linear mixed-effects sensitivity analyses. Supplementary Table S4 provides the ranked standardized sensory descriptors. The Python version 3.10 scripts used for corpus preprocessing, statistical analysis, and text mining are available from the corresponding author upon reasonable request.
