# Supplementary material for: Text Mining Analysis of Q-Grader Sensory Descriptors in Specialty Coffee Under Accelerated Storage Conditions
Source: Foods. 2026 Aug 5;15(15):2756. doi: 10.3390/foods15152756 (PMC13465152; doi:10.3390/foods15152756)
Supplement: Supplementary file 1 [file foods-15-02756-s001.zip › Supplementary Table S2.pdf]

Supplementary Table S2

Table S2. Quantitative validation metrics for candidate Latent Dirichlet Allocation (LDA) models used to determine the optimal number of latent topics.

Number of topics (k)	NPMI coherence	Topic stability	Topic diversity	Perplexity
2	0.062	0.91	0.77	65.7
3	0.074	0.48	0.68	67.9
4	0.072	0.47	0.58	69.9
5	0.07	0.44	0.52	72
6	0.069	0.42	0.54	74
7	0.079	0.42	0.52	73.5
8	0.071	0.53	0.43	76
9	0.096	0.35	0.44	77.8
10	0.07	0.42	0.42	78.6

Note. Candidate LDA models containing between two and ten latent topics were compared using four complementary validation metrics: normalized pointwise mutual information (NPMI) coherence, topic stability, topic diversity, and perplexity. Higher coherence, stability, and diversity values indicate better semantic consistency and reproducibility, whereas lower perplexity indicates improved predictive performance. The optimal two-topic model was selected because it provided the best overall balance among the four validation criteria. The composite validation framework integrated all four metrics simultaneously to guide model selection rather than relying on a single optimization criterion.
