# Supplementary material for: Text Mining Analysis of Q-Grader Sensory Descriptors in Specialty Coffee Under Accelerated Storage Conditions
Source: Foods. 2026 Aug 5;15(15):2756. doi: 10.3390/foods15152756 (PMC13465152; doi:10.3390/foods15152756)
Supplement: Supplementary file 1 [file foods-15-02756-s001.zip › Supplementary Table S3.pdf]

## Supplementary Table S3

**Table S3.** Fixed-effect estimates and model-fit statistics from the linear mixed-effects sensitivity models.

### A. Complete dataset (Packaging–product configurations A–H)

| Fixed effect | β | SE | 95% CI | p-value |
|---|---|---|---|---|
| Intercept (PPC A) | 84.296 | 0.141 | 84.019 to 84.573 | <0.001 |
| PPC B | 1.365 | 0.143 | 1.083 to 1.646 | <0.001 |
| PPC C | 1.521 | 0.143 | 1.240 to 1.802 | <0.001 |
| PPC D | 1.583 | 0.143 | 1.302 to 1.864 | <0.001 |
| PPC E | −1.731 | 0.143 | −2.012 to −1.450 | <0.001 |
| PPC F | −0.146 | 0.143 | −0.427 to 0.135 | 0.309 |
| PPC G | −0.003 | 0.143 | −0.285 to 0.278 | 0.981 |
| PPC H | 0.045 | 0.143 | −0.236 to 0.326 | 0.753 |
| Storage temperature (°C) | 0.053 | 0.004 | 0.044 to 0.061 | <0.001 |
| Storage stage | −0.521 | 0.032 | −0.584 to −0.458 | <0.001 |

### B. Green coffee (Packaging–product configurations A–C)

| Fixed effect | β | SE | 95% CI | p-value |
|---|---|---|---|---|
| Intercept (PPC A) | 84.296 | 0.14 | 84.022 to 84.571 | <0.001 |
| PPC B | 1.365 | 0.141 | 1.088 to 1.641 | <0.001 |
| PPC C | 1.521 | 0.141 | 1.245 to 1.797 | <0.001 |
| Storage temperature (°C) | 0.023 | 0.007 | 0.009 to 0.037 | 0.001 |
| Storage stage | −0.369 | 0.051 | −0.470 to −0.269 | <0.001 |

### C. Roasted ground coffee (Packaging–product configurations E–H)

| Fixed effect | β | SE | 95% CI | p-value |
|---|---|---|---|---|
| Intercept (PPC E) | 80.982 | 0.122 | 80.742 to 81.222 | <0.001 |
| PPC F | 1.585 | 0.123 | 1.344 to 1.827 | <0.001 |
| PPC G | 1.728 | 0.123 | 1.486 to 1.969 | <0.001 |
| PPC H | 1.776 | 0.123 | 1.535 to 2.018 | <0.001 |
| Storage temperature (°C) | 0.078 | 0.005 | 0.068 to 0.089 | <0.001 |
| Storage stage | −0.691 | 0.039 | −0.767 to −0.614 | <0.001 |

**Note.** Linear mixed-effects models were fitted using cup score as the response variable; packaging–product configuration (PPC), storage temperature, and storage stage as fixed effects; and Q Arabica Grader as a random intercept. Models were estimated by maximum likelihood (ML) and included as sensitivity analyses to evaluate whether persistent between-grader variability influenced the principal interpretation obtained from the repeated-measures analyses. β denotes the estimated fixed-effect coefficient; SE, standard error; CI, confidence interval; LogLik, log-likelihood; AIC, Akaike Information Criterion; BIC, Bayesian Information Criterion; ICC, conditional intraclass correlation coefficient. The reference configuration was PPC A for the complete and green-coffee models and PPC E for the roasted ground-coffee model.
