# Supplementary material for: Text Mining Analysis of Q-Grader Sensory Descriptors in Specialty Coffee Under Accelerated Storage Conditions
Source: Foods. 2026 Aug 5;15(15):2756. doi: 10.3390/foods15152756 (PMC13465152; doi:10.3390/foods15152756)
Supplement: Supplementary file 1 [file foods-15-02756-s001.zip › Supplementary Table S4.pdf]

Supplementary Table S4

Table S4. Top standardized sensory descriptors ranked by mean term frequency–inverse document frequency according to the complete corpus, accelerated storage temperature, and packaging–product configuration.

Grouping	Level	Rank	Descriptor	Mean TF-IDF
Overall	All	1	brown_sugar	0.134112014
Overall	All	2	chocolate	0.116137782
Overall	All	3	carob	0.105707411
Overall	All	4	cardboard	0.102870836
Overall	All	5	caramel	0.10209503
Overall	All	6	malt	0.099331165
Overall	All	7	floral	0.093703615
Overall	All	8	honey	0.08729092
Overall	All	9	nut	0.085685486
Overall	All	10	orange	0.07897437
Overall	All	11	sweet	0.073595227
Overall	All	12	maple	0.073397934
Overall	All	13	stale	0.072349897
Overall	All	14	plum	0.071734785
Overall	All	15	legume	0.068880217
Temperature	40 °C	1	brown_sugar	0.1518812
Temperature	40 °C	2	chocolate	0.120454502
Temperature	40 °C	3	nut	0.111522818
Temperature	40 °C	4	cardboard	0.106194726
Temperature	40 °C	5	floral	0.09546974
Temperature	40 °C	6	caramel	0.094396815
Temperature	40 °C	7	malt	0.094273185
Temperature	40 °C	8	carob	0.092234744
Temperature	40 °C	9	green_apple	0.088207578
Temperature	40 °C	10	stale	0.083059228
Temperature	40 °C	11	honey	0.081697244
Temperature	40 °C	12	red_apple	0.078532233
Temperature	40 °C	13	orange	0.07607489
Temperature	40 °C	14	sweet	0.075471642
Temperature	40 °C	15	woody	0.073746206
Temperature	50 °C	1	brown_sugar	0.143223329
Temperature	50 °C	2	caramel	0.117127881
Temperature	50 °C	3	chocolate	0.114376513
Temperature	50 °C	4	cardboard	0.110227486
Temperature	50 °C	5	carob	0.108835289
Temperature	50 °C	6	malt	0.107754573
Temperature	50 °C	7	floral	0.101039905
Temperature	50 °C	8	plum	0.091111677
Temperature	50 °C	9	orange	0.089996962
Temperature	50 °C	10	honey	0.088895995
Temperature	50 °C	11	maple	0.087916153
Temperature	50 °C	12	nut	0.0773835

Temperature	50 °C	13	sweet	0.076655116
Temperature	50 °C	14	red_apple	0.065669795
Temperature	50 °C	15	toffee	0.064812006
Temperature	60 °C	1	carob	0.1160522
Temperature	60 °C	2	chocolate	0.11358233
Temperature	60 °C	3	brown_sugar	0.107231514
Temperature	60 °C	4	malt	0.095965738
Temperature	60 °C	5	caramel	0.094760394
Temperature	60 °C	6	cardboard	0.092190296
Temperature	60 °C	7	honey	0.09127952
Temperature	60 °C	8	floral	0.0846012
Temperature	60 °C	9	apple	0.081697039
Temperature	60 °C	10	maple	0.080580486
Temperature	60 °C	11	dry	0.076919664
Temperature	60 °C	12	legume	0.074579578
Temperature	60 °C	13	vanilla	0.072195825
Temperature	60 °C	14	orange	0.070851257
Temperature	60 °C	15	peach	0.070666565
Configuration	A	1	green_tea	0.228384628
Configuration	A	2	brown_sugar	0.203015559
Configuration	A	3	astringent	0.183385873
Configuration	A	4	malt	0.142085861
Configuration	A	5	herbal	0.13912405
Configuration	A	6	green_apple	0.118870512
Configuration	A	7	orange	0.117175136
Configuration	A	8	dry	0.114016529
Configuration	A	9	chocolate	0.10804968
Configuration	A	10	hazelnut	0.098439496
Configuration	A	11	bitter	0.097753865
Configuration	A	12	carob	0.095467498
Configuration	A	13	nut	0.094667909
Configuration	A	14	woody	0.093307478
Configuration	A	15	plum	0.09094483
Configuration	B	1	floral	0.127333873
Configuration	B	2	brown_sugar	0.125275758
Configuration	B	3	manzanilla	0.119868638
Configuration	B	4	peach	0.112352796
Configuration	B	5	chocolate	0.112161313
Configuration	B	6	caramel	0.108198201
Configuration	B	7	nut	0.107008274
Configuration	B	8	almond	0.099471256
Configuration	B	9	sweet	0.098301279
Configuration	B	10	orange	0.091737481
Configuration	B	11	pintona	0.087756299
Configuration	B	12	red_apple	0.087160631
Configuration	B	13	lemon	0.087050467
Configuration	B	14	fruta	0.086054552
Configuration	B	15	cereal	0.082909841
Configuration	C	1	floral	0.157401601

Configuration	C	2	hazelnut	0.152735605
Configuration	C	3	peach	0.13359273
Configuration	C	4	red_apple	0.129051486
Configuration	C	5	chocolate	0.125119121
Configuration	C	6	orange	0.123488122
Configuration	C	7	manzanilla	0.120525408
Configuration	C	8	honey	0.113810369
Configuration	C	9	brown_sugar	0.105530595
Configuration	C	10	malt	0.099190958
Configuration	C	11	caramel	0.0990912
Configuration	C	12	jugoso	0.096039325
Configuration	C	13	vanilla	0.091315476
Configuration	C	14	apple	0.090966858
Configuration	C	15	almond	0.089154983
Configuration	D	1	honey	0.231166108
Configuration	D	2	floral	0.179735369
Configuration	D	3	red_apple	0.156647008
Configuration	D	4	vanilla	0.142002029
Configuration	D	5	brown_sugar	0.140604256
Configuration	D	6	sweet	0.139070291
Configuration	D	7	manjar	0.137325344
Configuration	D	8	caramel	0.136808603
Configuration	D	9	chocolate	0.116528571
Configuration	D	10	maple	0.115792641
Configuration	D	11	peach	0.115187573
Configuration	D	12	malt	0.113416759
Configuration	D	13	stale	0.106677392
Configuration	D	14	carob	0.104951969
Configuration	D	15	cana	0.095812151
Configuration	E	1	cardboard	0.284425759
Configuration	E	2	legume	0.236949169
Configuration	E	3	liviano	0.170946596
Configuration	E	4	oxidized	0.163068122
Configuration	E	5	stale	0.130356951
Configuration	E	6	brown_sugar	0.12878367
Configuration	E	7	papa	0.111476282
Configuration	E	8	chocolate	0.108687195
Configuration	E	9	roasted_peanut	0.107906405
Configuration	E	10	dry	0.097807397
Configuration	E	11	toasted_bread	0.095414662
Configuration	E	12	nut	0.094956967
Configuration	E	13	plum	0.077234091
Configuration	E	14	black_tea	0.074840883
Configuration	E	15	dark_chocolate	0.071899579
Configuration	F	1	cardboard	0.204464269
Configuration	F	2	malt	0.16495513
Configuration	F	3	carob	0.147299828
Configuration	F	4	maple	0.13392283
Configuration	F	5	chocolate	0.131605784

Configuration	F	6	caramel	0.116256919
Configuration	F	7	legume	0.116136437
Configuration	F	8	toasted_bread	0.106345584
Configuration	F	9	plum	0.10411539
Configuration	F	10	nut	0.102970936
Configuration	F	11	honey	0.101889495
Configuration	F	12	orange	0.100955152
Configuration	F	13	oxido	0.094372087
Configuration	F	14	floral	0.092269602
Configuration	F	15	brown_sugar	0.089291589
Configuration	G	1	cardboard	0.174754561
Configuration	G	2	carob	0.149008114
Configuration	G	3	brown_sugar	0.146981713
Configuration	G	4	chocolate	0.125273182
Configuration	G	5	maple	0.109257593
Configuration	G	6	caramel	0.105393354
Configuration	G	7	honey	0.105070357
Configuration	G	8	meloso	0.09432954
Configuration	G	9	bitter	0.090290679
Configuration	G	10	malt	0.088936563
Configuration	G	11	plum	0.088905679
Configuration	G	12	stale	0.086167781
Configuration	G	13	nut	0.085315669
Configuration	G	14	floral	0.084649151
Configuration	G	15	sweet	0.083811875
Configuration	H	1	carob	0.145787452
Configuration	H	2	cardboard	0.134728957
Configuration	H	3	stale	0.134640091
Configuration	H	4	brown_sugar	0.133412973
Configuration	H	5	orange	0.124856178
Configuration	H	6	caramel	0.115983296
Configuration	H	7	malt	0.111831397
Configuration	H	8	toffee	0.106496573
Configuration	H	9	chocolate	0.101677407
Configuration	H	10	liviano	0.091908342
Configuration	H	11	legume	0.090187484
Configuration	H	12	nut	0.084917924
Configuration	H	13	herbal	0.082412357
Configuration	H	14	woody	0.082098321
Configuration	H	15	plum	0.07924121

Note. TF–IDF scores were calculated from the normalized document–term matrix. Higher values indicate descriptors that were comparatively more representative of the corresponding corpus, storage temperature, or packaging–product configuration, rather than descriptors with the highest absolute frequency alone. The overall corpus and each experimental grouping are presented with the 15 descriptors showing the highest mean TF–IDF values. PPCs A–H correspond to the packaging–product configurations defined in Table 1. Compound descriptors were retained as single analytical units using underscore notation.
